# Small DUF1127 proteins regulate bacterial phosphate metabolism through protein–protein interactions with the sensor kinase PhoR

**DOI:** 10.1093/femsml/uqaf023

**Published:** 2025-09-18

**Authors:** Donata C L E Remme, Lea-Janina Tilg, Yvonne Pfänder, Jing Yuan, Franz Narberhaus

**Affiliations:** Microbial Biology, Ruhr University Bochum, 44780 Bochum, Germany; Microbial Biology, Ruhr University Bochum, 44780 Bochum, Germany; Microbial Biology, Ruhr University Bochum, 44780 Bochum, Germany; Max Planck Institute for Terrestrial Microbiology and Center for Synthetic Microbiology, 35043 Marburg, Germany; Microbial Biology, Ruhr University Bochum, 44780 Bochum, Germany

**Keywords:** DUF1127, small proteins, bacteria, phosphate uptake, protein interaction, sensor kinase

## Abstract

The domain of unknown function 1127 (DUF1127) is widely distributed among bacteria, often in proteins shorter than 50 amino acids. In the plant pathogen *Agrobacterium tumefaciens*, the absence of three small DUF1127 proteins leads to a range of phenotypic changes. In this study, we investigated the role of these small DUFs in phosphate acquisition. Upregulation of phosphate transport systems in the triple mutant resulted in increased phosphate uptake, polyphosphate accumulation, and growth defects. Using Far–Western dot blots, pulldown experiments, and the bacterial two-hybrid system, we identified a direct interaction between the small DUFs and the sensor kinase PhoR, which regulates phosphate metabolism together with the response regulator PhoB. Complementation studies revealed that DUF1127 proteins from *Sinorhizobium meliloti, Rhodobacter sphaeroides*, and *Escherichia coli* could restore the phenotypes in the *A. tumefaciens* triple mutant. Notably, an *E. coli* mutant lacking YjiS, the sole DUF1127 protein in this species, showed upregulated expression of phosphate uptake genes and accelerated phosphate uptake. Furthermore, we provide evidence for an interaction between YjiS and *E. coli* PhoR, suggesting that DUF1127-containing proteins may share a conserved regulatory function across different bacterial species. These findings provide new insights into the function of small DUF1127 proteins, demonstrating that they can act through protein–protein interactions.

## Teaser

Conserved small proteins modulate bacterial phosphate homeostasis by interacting with the sensor kinase PhoR of the phosphate uptake system.

## Introduction

More than 20% of the proteins across all domains of life contain conserved domains of unknown function (DUFs), with nearly half of them (43%) occurring in bacteria. This amounts to ~2700 different bacterial DUFs, many of them with essential functions (Goodacre et al. 2013, Price et al. 2018). Unraveling their critical biological roles, or “de-DUFing” the DUFs, has become an exciting area of research (Mudgal et al. 2015). Notably, many DUF-containing proteins are small, often shorter than 100 or even 50 amino acids, and it is becoming increasingly clear that the role of these proteins in bacterial and archaeal physiology has been underestimated (Jordan et al. 2023, Burton et al. 2024). The sORFdb database, which catalogs small open reading frames and small proteins from bacteria, reports over five million nonredundant small proteins (Hahnfeld et al. 2025).

Amongst the largest bacterial protein families is DUF1127, with nearly 25 000 members in the UniProt database (UniProt Consortium 2004). This domain is typically arginine-rich, positively charged, and primarily found in alphaproteobacteria and gammaproteobacteria (Kraus et al. 2020). While alphaproteobacteria often encode multiple DUF1127 proteins, gammaproteobacteria tend to harbor only one. In *Escherichia coli* and *Salmonella*, this 54-amino acid protein is known as YjiS (Sibley and Raleigh 2004). Despite their small size, DUF1127 proteins can have a significant impact on bacterial metabolism and behavior (Alakavuklar and Fuqua 2020). In *Salmonella, yjiS* expression is highly induced upon host invasion and plays a role in bacterial escape from macrophages (Westermann et al. 2016, Venturini et al. 2025). In *Brucella abortus*, the three DUF1127 proteins have been linked to fucose utilization (Budnick et al. 2018). In *Rhodobacter sphaeroides*, the DUF1127 protein CcaF1 modulates C1 metabolism, the oxidative stress defense, and photosynthetic complex formation (Billenkamp et al. 2015, Grützner et al. 2023). The plant pathogen *Agrobacterium tumefaciens* encodes seven DUF1127-containing proteins: three short ones (short DUF1127 proteins; SDPs) of 47 or 48 amino acids, and four longer ones (long DUF1127 proteins) ranging from 72 to 102 amino acids. Deleting all three SDPs caused drastic transcriptomic and phenotypic changes, implicating these proteins in biofilm formation as well as phosphate and carbon metabolism (Kraus et al. 2020). Similarly, DUF1127 deletion in *Vibrio alginolyticus* affected biofilm formation (Feng et al. 2024).

But how can small DUF1127 proteins exert such a broad influence on bacterial physiology and lifestyle decisions? The *R. sphaeroides* CcaF protein has been documented as an RNA-binding protein involved in sRNA maturation and RNA turnover, which might explain its widespread role in the physiology of this photosynthetic bacterium (Grützner et al. 2021). However, this contrasts with recent findings on *Salmonella* YjiS, where no evidence for RNA binding was found. Instead, YjiS interacts with various proteins, including the sensor kinase SsrA (Venturini et al. 2025).

Our study aimed to understand how the three *Agrobacterium* SDPs influence phosphate metabolism, building on the finding that phosphate acquisition genes like *pstS* are upregulated >100-fold in the triple SDP mutant (Kraus et al. 2020). Consistent with this upregulation, we observed that the mutant strain took up phosphate more rapidly than the wild-type (WT). To investigate the underlying mechanism, we tested whether the SDPs physically interact with components of the phosphate-specific transporter (PstS, PstC, PstA, PstB) or the regulatory system, which includes the two-component system PhoR–PhoB and the accessory regulator PhoU (Danhorn et al. 2004, Santos-Beneit 2015). Notably, three independent assays consistently demonstrated an interaction between the *A. tumefaciens* SDPs and the sensor kinase PhoR.

Furthermore, we found that DUF1127 proteins from *Sinorhizobium meliloti, R. sphaeroides*, and *E. coli* could restore phenotypes of the *A. tumefaciens* triple SDP mutant, suggesting a conserved function of DUF1127 proteins across bacterial species. Interestingly, the *E. coli yjiS* mutant also exhibited accelerated phosphate uptake and YjiS bound to PhoR, supporting the idea that the regulation of phosphate acquisition via DUF1127–PhoR interaction is a conserved mechanism.

## Material and methods

### Bacterial strains and plasmids

A detailed list of all bacterial strains used in this study is provided in Table S1. Plasmids and cloning strategies are described in Table S2, and the oligonucleotides employed are listed in Table S3.

### Deletion mutants

The method of constructing a marker-less deletion mutant of *pstS* is described in Kraus et al. (2020). The primers for the polymerase chain reaction (PCR) amplification are listed in Table S3.

### Culture conditions and media

*Agrobacterium tumefaciens* strains were inoculated at an OD_600_ of 0.1 in YEB medium (0.1% yeast extract, 0.5% beef extract, 0.5% peptone, 0.5% sucrose, and 1 mM MgSO_4_). For the growth curves and phosphate measurements, *A. tumefaciens* strains were inoculated at an OD_600_ of 0.1 in minimal medium (MM) (20.8 mM K₂HPO_4_, 15.1 mM NaH₂PO_4_, 18.7 mM NH_4_Cl, 2 mM KCl, 50 mM MES, 50 mM KOH, 0.1 mM CaCl₂, 15.5 mM MgSO_4_, 0.01 mM FeSO_4_, and 0.5% sucrose, pH 7.0) at 30°C and 130 rpm.

For the complementation studies, genes were expressed from the pSRK vector (Table S2) using the native *sdp1* promoter from *A. tumefaciens* (Table S4), which allowed constitutive expression without induction.

*Escherichia coli* strains were inoculated for each experiment at an OD_600_ of 0.1 in trehalose MM (20.8 mM K₂HPO_4_, 15.1 mM NaH₂PO_4_, 18.7 mM NH_4_Cl, 2 mM KCl, 50 mM MES, 50 mM KOH, 0.1 mM CaCl₂, 15.5 mM MgSO_4_, 0.01 mM FeSO_4_, and 0.5% trehalose, pH 7.0) at 37°C and 130 rpm.

For the protein purifications of the ^His-SUMO^SDP proteins, His-SUMO, both ^Strep^PhoR proteins, ^Strep^PhoU, ^Strep^PstS and ^Strep^PstB the *E. coli* BL21 strain carrying the plasmid was inoculated at an OD_600_ of 0.1 in LB medium at 37°C (10 g/l tryptone, 5 g/l yeast extract and 10 g/l NaCl). After reaching an OD_600_ of 0.5, expression was induced by adding 1.6 mM IPTG or 200 µg/ml AHT (anhydrotetracycline). The cells were harvested 3 h after induction by centrifugation and washed with *A. dest*. (*Aqua destillata*). Then the cell pellets were stored at −20°C. For the protein purification of PhoB, the *E. coli* strain containing the corresponding plasmid was inoculated at an OD_600_ of 0.1 in TB medium (12 g/l tryptone, 24 g/l yeast extract, and 0.44% glycerol were mixed with 900 ml water and autoclaved. Then, 100 ml of a solution containing 0.17 M KH₂PO_4_ and 0.72 M K₂HPO_4_ was added to adjust the volume to 1 l). At an OD_600_ of 0.5, expression was induced by adding 200 µg/ml AHT. The cells were cultivated overnight at 16°C, harvested, and washed with distilled water. The cell pellets were stored at −20°C.

### PolyP isolation

For polyphosphate isolation, cell pellets were harvested at the required time point and adjusted to an OD_600_ of four. After harvesting, the pellets were stored overnight at −20°C. The next day, the cells were resuspended in 500 µl of cold AE buffer (50 mM sodium acetate and 10 mM EDTA). Then, 40 µl of SDS (10%) and 300 µl of phenol were added. The samples were incubated at 65°C for 5 min and then on ice for 1 min. In the next step, 300 µl of chloroform was added, and the samples were centrifuged at 13 000 rpm at room temperature for 2 min. The upper phase was transferred to a new tube and incubated with 2 µl of RNase (stock concentration of 10 mg/ml) and 2 µl of DNase (stock concentration of 10 mg/ml) for 1 h at 37°C. Afterward, the sample was transferred to a new tube. Additionally, 40 µl of sodium acetate (3 M) and 1 ml of cold ethanol (100%) were added. The precipitation occurred overnight at −20°C. The next day, the sample was centrifuged for 20 min at 4°C, and the supernatant was removed. The sample was washed with ethanol (70%) and centrifuged again for 5 min at 4°C. The supernatant was removed, and the pellets were dried. Finally, the dried pellets were dissolved in 1 ml of water and stored at −20°C until polyphosphate measurement.

To measure the polyphosphate content in the samples, 150 µl of the polyP extracts were mixed with 5x ScPPX1 reaction buffer (100 mM Tris-HCl, 25 mM MgCl_2_, 250 mM ammonium acetate, pH 7.5). The samples were then incubated at 37°C for 15 min with 1 µg of purified PPX protein from *E. coli* in a total volume of 200 µl. The phosphate was measured as described in the next section. For this experiment, 100 µl of the undiluted polyphosphate samples were used per well.

### Phosphate measurement

The method for measuring phosphate content in the supernatant was adapted from Murphy and Riley (1962) and Anschutz and Deborde (2016). Samples of the culture supernatant were collected at different time points. The supernatant was sterilized using a 0.2 µm pore size filter and stored at 4°C until the next day. Each sample was diluted 1:16 with water. Then, 100 µl of each diluted sample was added to a 96-well plate, with water used as a blank. Next, MR-Mix II was freshly prepared by adding 2 ml of ascorbic acid (613 mM) to 8 ml of MR-Mix I (containing 16.1 M sulfuric acid, 6.1 mM ammonium molybdate solution, and 513 µM antimony solution). To each sample, 10 µl of MR-Mix II was added. The samples were incubated at room temperature for up to 5 min. The optical density was then measured at 885 nm.

### Cell aggregate assay

The cell aggregation assay was performed as described in Kraus et al. (2020). Photos were taken after 24 h.

### Foaming assay

*Agrobacterium tumefaciens* strains were inoculated in YEB medium with or without antibiotics and incubated overnight at 30°C at 180 rpm. The next day, 100 ml of the overnight culture was used to inoculate 1-l baffled flasks to an OD_600_ of 0.1. The cultures were grown at 30°C with shaking at 180 rpm. Photos were taken after 24 h.

### Determination of cell length by microscopy

Cells of *A. tumefaciens* were cultivated for 24 h in YEB medium. The cells were then harvested by centrifugation (13 000 rpm) and washed with phosphate-buffered saline (PBS) buffer. The OD_600_ was adjusted to 2 in 1 ml. A total of 10 µl of the samples were placed on agarose slides covered with 700 µl of 1.5% agarose in PBS. For microscopy, an Olympus BC-51 fluorescence microscope was used, and cell length was measured using the VisiView software by Visitron Systems GmbH.

### Isolation of RNA and Northern blot analyses

For the RNA isolation from *A. tumefaciens* the cells were cultivated in YEB medium, and samples were taken after 8 h of incubation at 30°C. For *E. coli*, the strains were cultivated in MM with trehalose and samples were taken after 4, 8, and 24 h. For both bacteria, 10 ml of culture was harvested. RNA isolation was then performed as described by Wilms et al. (2011). A total of 5 µg of RNA per sample were used and adjusted to a total volume of 10 µl. Then, 5 µl of loading dye (NEB) was added to each sample. The RNA was separated on a 1.2% MOPS-agarose gel (20 mM MOPS, 5 mM Na-acetate, and 1 mM EDTA, 8% formaldehyde; pH 7.0). After separation, the RNA was blotted using either capillary blotting or electroblotting (Higo et al. 2002, Wilms et al. 2011). After blotting, the RNA was fixed to the membrane by placing it on a UV table for 3 min. The washing of the membrane and the detection of the samples were performed as described in Wilms et al. (2011). The primers used for the probes in the Northern blots are listed in Table S3.

### qRT-PCR

Quantitative real-time PCR (qRT-PCR) experiments were performed as described in Schmidt et al. (2024).

### Purification of DUF1127^His-SUMO^ proteins and the His-SUMO tag

For the purification of the DUF1127 protein with a His-SUMO tag or the purification of the tag alone, the cells were lysed using the B-PER Complete Bacterial Protein Extraction Reagent (Thermo Fisher Scientific) according to the manufacturer’s instructions. The samples were centrifuged for 10 min at 4°C at 8960 *g*, and the supernatant was filtered through a 0.2 µm pore size filter. The filtered supernatant was loaded onto a Ni-NTA column and incubated at 4°C for 1 h at 130 rpm. After this, the column was allowed to stand for 10 min to let the resin settle. The flowthrough was collected. The column was then washed with 10 ml of wash buffer I (20 mM HEPES, 50 mM imidazole, 500 mM NaCl, and 10% glycerol, pH 8), followed by 10 ml of wash buffer II (20 mM HEPES, 50 mM imidazole, 300 mM NaCl, and 10% glycerol, pH 8), and finally wash buffer III (20 mM HEPES, 50 mM imidazole, 150 mM NaCl, and 10% glycerol, pH 8). The bound proteins were eluted with 5 × 1 ml of elution buffer (20 mM HEPES, 250 mM imidazole, 150 mM NaCl, and 10% glycerol).

### Protein purification of Strep-tagged proteins

For the purification of the PhoR protein with a Strep-Tag, the cells were resuspended in PBS buffer (4 mM KH₂PO_4_, 16 mM Na₂HPO_4_, 115 mM NaCl) and lysed using a French Press, three to four times at a pressure setting of 900 psi. The samples were centrifuged for 10 min at 4°C at 8960 *g*, and the supernatant was filtered through a 0.2 µm pore size filter. The filtered supernatant was separated via ultracentrifugation for 1.5 h at 4°C (200 000 *g*). The supernatant was removed, and the membrane pellets were solubilized in 2 ml solubilization buffer (50 mM Tris, 100 mM NaCl, 2% Triton X-100, pH 8) overnight under stirring at 4°C. Then, the solution was loaded onto a Strep-Tactin resin column and incubated at 4°C for 1 h at 130 rpm. After this, the column was allowed to stand for 10 min to let the resin settle, and the flowthrough was collected. The column was then washed five times with 2 ml of wash buffer from IBA Life Sciences. The bound proteins were eluted in three steps using 800, 1400, and 800 µl of elution buffer (IBA Life Sciences).

### Far–Western dot blots

The Far–Western dot blots were performed as described in Möller et al. (2023). EveryBlot Blocking Buffer (Bio-Rad) was used as the blocking buffer, and phosphate-buffered saline with tween (PBST) (4 mM KH₂PO_4_, 16 mM Na₂HPO_4_, 115 mM NaCl and 0.3% tween) was used as the washing buffer. As controls the strep tagged proteins were spotted alone on the membrane and detected with an antibody against the strep tag to verify the presence of the protein. The same was done with the His-SUMO tagged protein with an antibody against the His tag.

### Pulldown assay

For the pulldown assay, 125 µg of bait protein and 125 µg of prey protein were mixed in 500 mM KH_2_PO_4_ to a total volume of 500 µl and incubated for 1 h on ice at 30 turns per minute on a laboratory tilting table. A total of 100 µl of the mixture were separated and stored at −20°C as the mixture sample. The remaining 400 µl of the mixture were then loaded onto a column with Strep-Tactin resin (IBA Life Sciences) or Ni-NTA resin (Qiagen) and incubated there for 30 min at 4°C. The flowthrough was collected. Afterward, the column was washed three times with 400 µl of the wash buffer from IBA Life Sciences for the strep resin. In the case of the Ni-NTA resin, 400 µl each of wash buffer I, wash buffer II, and wash buffer III were added to the column. All wash fractions were collected and kept for analysis. In the final step, 200 µl of the elution buffer from IBA Life Sciences was used for the Strep-Tactin column, or 200 µl of the elution buffer for the Ni-NTA column was used. To verify that the PhoR protein interacts with the SDPs and not with the His-SUMO tag alone, Pulldown experiments with the Strep tagged PhoR and the His-SUMO tag alone were performed.

### Bacteria-two-hybrid assay

The bacteria-two-hybrid assay was performed as described in Möller et al. (2023). As negative control the empty vectors were used and as positive controls the T18 and T25 domains fused on the fragments of the leucine zipper GCN4.

## Results

### Upregulation of the *pst* locus in the SDP triple mutant

*Agrobacterium tumefaciens* employs four distinct phosphor acquisition systems. One of these, the Pit system, functions as a constitutively expressed low-affinity permease. The remaining three systems are induced under phosphate-limited conditions. The Phn system is believed to transport phosphonates, the Ugp-system is specific for glycerol-3-phosphate, and the Pst system operates as a high-affinity ABC transporter essential for inorganic phosphate uptake (Schweizer et al. 1982, Gelvin 2003, Xu et al. 2012, Stasi et al. 2019).

**Figure 1.**
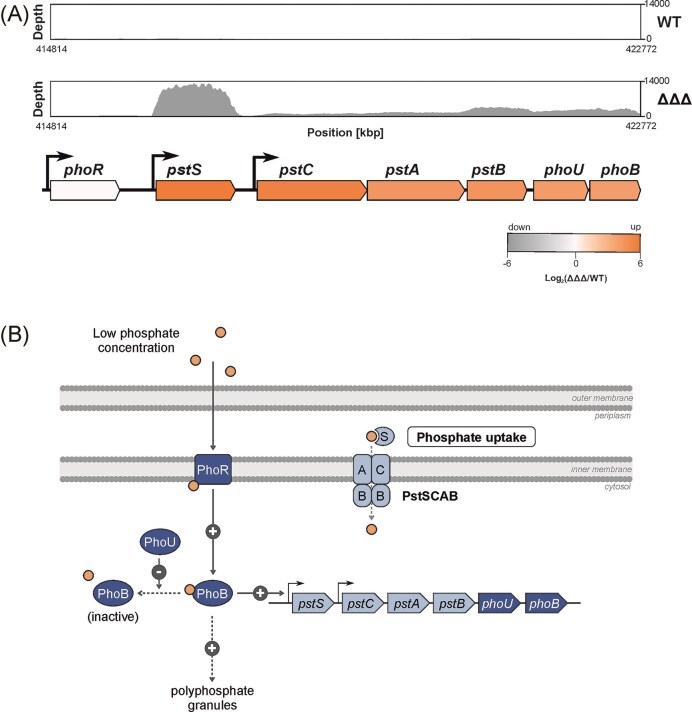
Upregulation of phosphate acquisition genes in the *A. tumefaciens* triple SDP mutant and schematic overview of the regulation and function of the Pst system. (A) RNA-sequencing profiles of the *pst* operon in the *A. tumefaciens* WT and ΔΔΔ strain showing upregulation of the *pst* gene cluster in the triple mutant. The results were taken from Kraus et al. (2020). The heatmap illustrates changes in gene expression as log₂ fold-change compared to the WT strain. The number of sequencing reads is given to the right. (B) Regulation and function of the Pst system, according to our knowledge in *E. coli* (Gardner and McCleary 2019). Under low phosphate conditions, the sensor kinase PhoR in the inner membrane is activated and phosphorylates the response regulator PhoB, which enhances transcription of phosphate uptake-related genes, including the ones coding for the PstSCAB transporter. PhoU interacts with PhoR and PstB to form a phosphate-signaling complex. For details, see Discussion.

Previous RNA sequencing experiments demonstrated that several phosphor uptake genes are significantly upregulated in the triple SDP mutant during the late exponential phase (Kraus et al. 2020). For example, *pstS*, which encodes the substrate-binding protein of the Pst system, was induced >100-fold. The genomic *pst* locus begins with *phoR* coding for a sensor kinase, whose expression remained unchanged in the absence of the three SDPs. In contrast, the subsequent six genes, in particular *pstS*, were strongly upregulated in the SDP mutant (Fig. 1A). Following *phoR*, the locus includes four *pst* genes that encode the structural components of the ABC transporter, as well as *phoU* and *phoB* (Fig. 1A and B). *phoU* is predicted to encode a regulatory protein, while the *phoB* gene product functions as a response regulator (Carmany et al. 2003, Bachhawat et al. 2005, Lamarche et al. 2008, Hsieh and Wanner 2010, Gardner et al. 2014, Santos-Beneit 2015). The transcriptome profile suggests the presence of promoters upstream of *phoR, pstS*, and *pstC* (Fig. 1A).

### Accelerated phosphate uptake and polyphosphate accumulation in the triple SDP mutant

**Figure 2.**
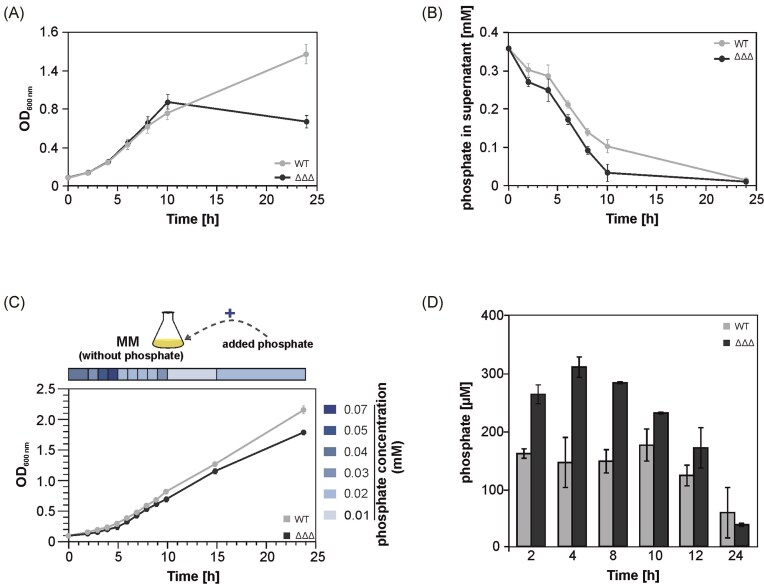
Phosphate-related phenotypes of the triple SDP mutant. (A) Growth curves of the *A. tumefaciens* WT strain and the triple SDP mutant (ΔΔΔ) in MM. The growth was measured in quintuplicates. (B) Phosphate concentration remaining in the supernatant over time for the WT and ΔΔΔ strains in MM. The phosphate uptake was measured in triplicates, with each measurement taken in technical duplicates. (C) Growth of the WT and ΔΔΔ strains in phosphate-depleted MM. Phosphate was added at specific time points in calculated amounts required for WT growth. The experiment was performed in biological duplicates. (D) Phosphate content isolated from polyphosphate granules in the WT and ΔΔΔ strains. The experiment was performed in biological duplicates and each measurement was done in technical duplicates. Standard deviations are indicated by error bars.

To assess the physiological consequences of enhanced *pst* gene expression, we examined phosphate-related phenotypes in the *A. tumefaciens* triple SDP mutant. As previously reported (Kraus et al. 2020), the mutant strain ceased growth after 10 h, whereas the WT continued growth (Fig. 2A). Phosphate uptake measurements revealed that the ΔΔΔ mutant took up phosphate significantly faster than the WT (Fig. 2B). While the WT required 7 h to take up half of the phosphate, the mutant only needed five and had almost all phosphate consumed after 10 h, which likely contributed to the growth arrest observed at this time point (Fig. 2A). Growth of the mutant strain in phosphate-free MM could be restored to WT level when defined amounts of phosphate were supplied sequentially (Fig. 2C). The required amounts were calculated based on phosphate uptake by the WT in the previous experiment (Fig. 2B). Overall, these results support the notion that upregulation of phosphate acquisition systems in the SDP mutant leads to accelerated phosphate depletion, ultimately causing its growth defect.

Since the mutant cannot utilize the accumulating intracellular phosphate for growth (Fig. 2A), we investigated whether it accumulates polyphosphate. Polyphosphate serves as an intracellular phosphate storage form, functioning as both an energy reservoir and phosphate reserve (Achbergerová and Nahálka 2011). In *E. coli*, polyphosphates contribute to bacterial fitness, stress resistance, and the regulation of transcription and translation. They also act as protein-folding scaffolds and function as primordial chaperones (McInerney et al. 2006, Gray et al. 2014, Gray and Jakob 2015, Guan and Jakob 2024). We found that the SDP mutant accumulates substantially more polyphosphate than the WT, particularly in the first 8 h of growth (Fig. 2D). These findings suggest that small DUF1127 proteins play a crucial role in regulating phosphate homeostasis in *A. tumefaciens*.

### Restoration of WT phenotypes by deletion of *pstS* in the triple SDP mutant

**Figure 3.**
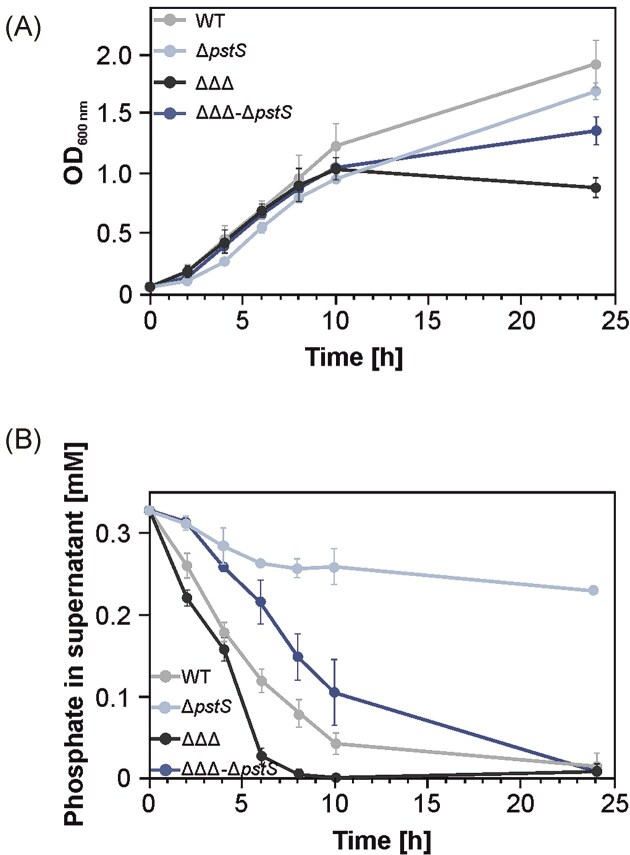
Phenotypic characterization of the *pstS* mutant and the triple SDP-*pstS* mutant. (A) Growth curves of the WT, ΔΔΔ, Δ*pstS* mutant, and quadruple mutant (Δ*pstS* mutant in the ΔΔΔ background) in YEB medium. Six replicates of each strain were measured. (B) Remaining phosphate concentration in the supernatant over time in the cultures grown in (A). Phosphate was measured in biological triplicates, with each measurement taken in technical duplicates. Standard deviations are indicated by error bars.

Notably, the observed phenotypes of both the WT and the triple deletion mutant were independent of the growth medium, as they were consistent in both MM (Fig. 2) and YEB medium (Fig. 3), highlighting the robustness of the phenotype across different conditions. To assess whether the altered phosphate uptake in the triple SDP mutant (ΔΔΔ) was linked to the upregulation of the Pst system, we deleted the *pstS* gene in both the WT and the ΔΔΔ backgrounds. The resulting *pstS* mutant and the ΔΔΔΔ (ΔΔΔ-Δ*pstS*) mutant exhibited slower growth than their respective parent strains. However, the ΔΔΔΔ strain ultimately reached a higher final OD than the ΔΔΔ strain (Fig. 3A). This is likely due to the reduced phosphate uptake in the absence of a functional Pst system (Fig. 3B), supporting the idea that the accelerated phosphate uptake in the triple SDP mutant is primarily dependent on the Pst transporter. Phosphate levels in the supernatant of the ΔΔΔΔ strain drop faster than in the Δ*pstS* mutant because other phosphate uptake systems are upregulated in the triple DUF1127 deletion mutant (Kraus et al. 2020).

### The SDPs interact with PhoR

We then investigated how the small DUF1127 proteins influence phosphate acquisition in *A. tumefaciens*. One possibility is that the SDPs physically interact with components of the Pho or Pst systems (Fig. 1B). To test this hypothesis, we employed three complementary approaches to determine protein–protein interactions.

First, we produced recombinant proteins and screened for interactions between the SDPs and various components of the Pho and Pst systems (PhoR, PhoU, PhoB, PstS, and PstB; Fig. 1B) using Far–Western dot blot experiments. Strep-tagged bait proteins were spotted onto a nitrocellulose membrane and incubated with His-SUMO-tagged SDPs. SDPs retained by the bait proteins were detected by immunoblotting with anti-His antibodies. As controls, all Strep-tagged bait proteins were successfully detected using an anti-Strep antibody, and His-SUMO-SDP3 spotted directly onto the membrane was detected by the anti-His antibody (Fig. S1A).

**Figure 4.**
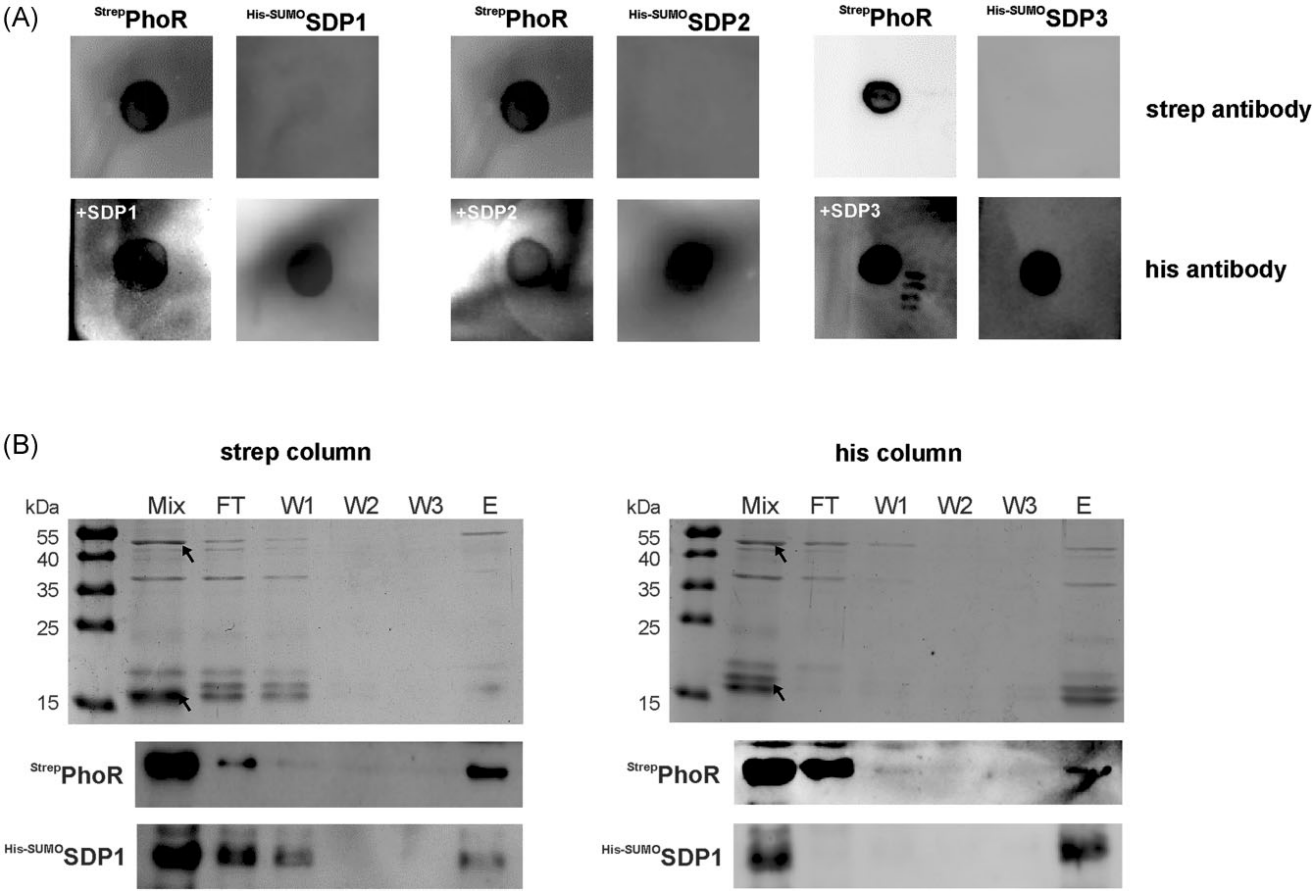
Protein–protein interactions between PhoR and small DUF1127 proteins shown by *in vitro* assays. (A) Far–Western dot blots using purified Strep-PhoR and His-SUMO-SDPs from *A. tumefaciens*. PhoR was spotted onto the membrane, which was then incubated with one of the SDPs. Signals indicating interaction were detected using an antibody against the His-SUMO tag of the SDPs. Interaction assays of SDP1 and SDP2 were carried out concurrently using the identical PhoR preparation on the same day and consequently, the same Strep antibody controls for PhoR detection are presented. (B) Pulldown experiments to examine the interaction between PhoR and SDP1. An interaction between these proteins was found regardless of whether His or Strep columns were used for protein purification. Coomassie-stained SDS–PAGE gels are shown on top and immunoblots at the bottom. Bands in the Coomassie-stained gel corresponding to those detected in the Western blot are indicated by arrows. FT = flow-through; W1–W3 = wash fractions 1–3; E = elution fraction.

SDP3, representing the three small DUF1127 proteins, did not bind to PhoB, PhoU, PstB, or PstS (Fig. S1A). Since each of the *Agrobacterium* SDPs can individually complement the phenotype of the triple SDP mutant (see next section and our unpublished results), we assume they share the same interaction profile and likewise do not bind to PhoB, PhoU, PstB, or PstS. Notably, all three SDPs interacted with the sensor kinase PhoR on the membrane (Fig. 4) suggesting a direct interaction between the DUF1127 proteins and PhoR.

To corroborate the PhoR–SDP interactions, we performed pulldown experiments using purified recombinant proteins as an additional *in vitro* approach. Consistent with the Far–Western blots (Fig. S1A), the pulldown assays revealed no interaction between PstS, PstB, PhoU, or PhoB and SDP3, which was again used as a representative of the SDPs (Fig. S1B). However, all three SDPs co-purified with PhoR in both pulldown (Strep-column binding PhoR) and reverse pulldown (His-column binding SDPs) experiments (Figs. 4 and S2). As a control, the His-SUMO tag alone did not bind Strep-PhoR (Fig. S2), confirming that the observed SDP–PhoR interactions can be attributed to the DUF1127 proteins and not the tag.

**Figure 5.**
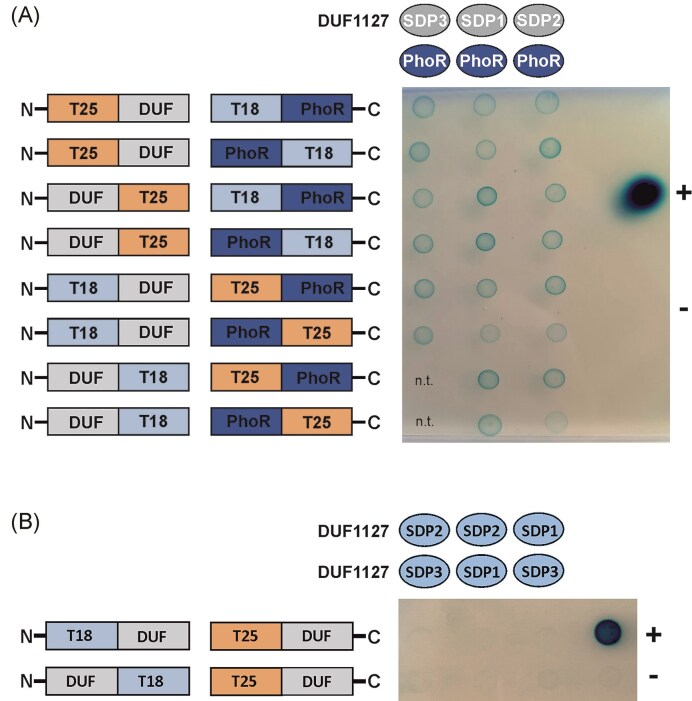
Bacterial two-hybrid analysis to determine protein–protein interactions between small *A. tumefaciens* DUF1127 proteins and PhoR in the *E. coli* reporter strain DHM1. (A) Interaction between the three SDPs and PhoR from *A. tumefaciens. Escherichia coli* cultures with the respective plasmids were spotted on agar plates containing X-Gal. (B) Absence of interaction between the three SDPs from *A. tumefaciens*. The positive control is spotted (+) is spotted above the negative control (−) on the right side of both plates. n. t. = not tested.

To further validate the interaction between the SDPs and PhoR, we employed the bacterial two-hybrid system as an *in vivo* approach. In this assay, the T25 or T18 domains of the *Bordetella pertussis* adenylate cyclase (CyaA) were fused either N- or C-terminally to *A. tumefaciens* SDPs or PhoR (Fig. 5). When the T18 and T25 domains are brought into proximity due to an interaction between the fused proteins, ATP is converted to cAMP, resulting in blue colonies on indicator plates (Karimova et al. 2005). Regardless of the fusion orientation, all SDPs interacted with PhoR as evidenced by the formation of blue colonies (Fig. 5A). Control experiments showed that the small DUF1127 proteins do not interact with each other (Fig. 5B).

In summary, our study provides three independent lines of evidence—Far–Western blotting, pulldown assays, and bacterial two-hybrid analysis—supporting a direct protein–protein interaction between the small *A. tumefaciens* DUF1127 proteins and the sensor kinase PhoR.

### Conserved function of small DUF1127 proteins across bacterial species

The DUF1127 domain is widely conserved across the bacterial kingdom, particularly within alphaproteobacteria and gammaproteobacteria. While alphaproteobacteria typically contain multiple DUF1127 proteins of varying sizes, gammaproteobacteria tend to harbor a single, smaller DUF1127 protein (Kraus et al. 2020).

**Figure 6.**
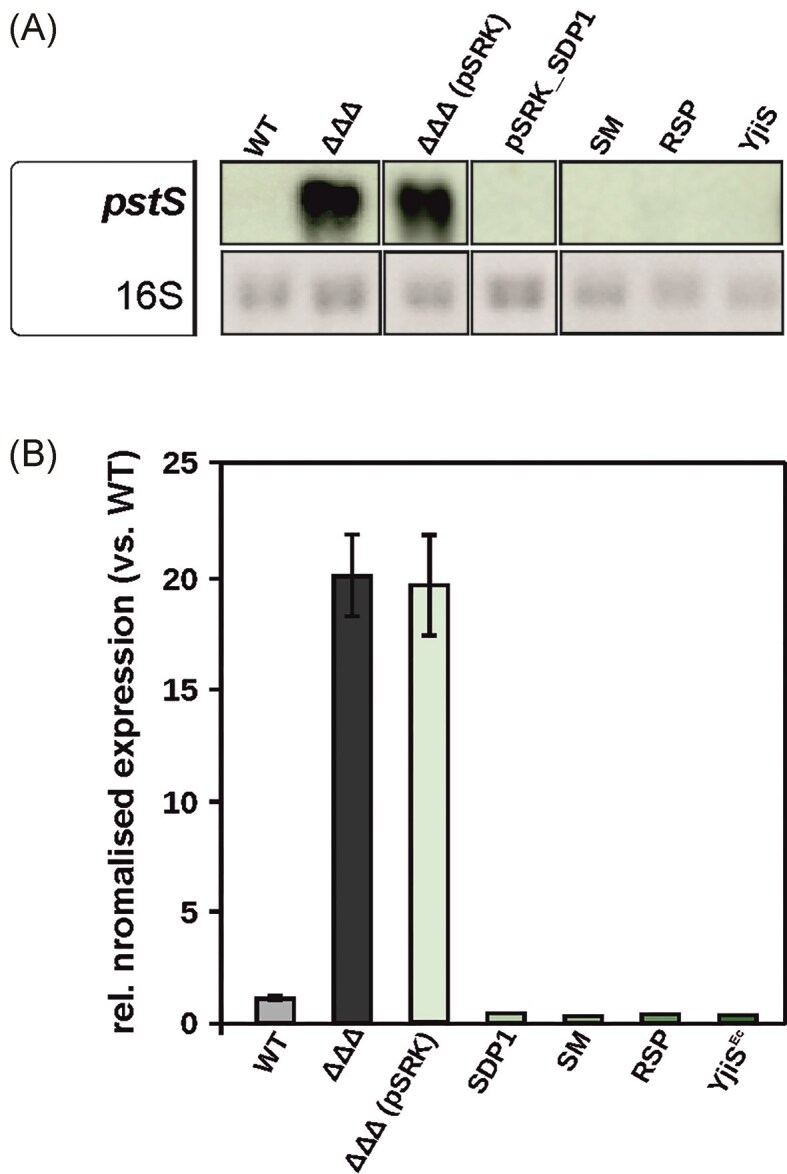
Various DUF1127 proteins can complement *pstS* regulation in the *A. tumefaciens* triple SDP mutant. (A) Northern blotting to assess the *pstS* transcription levels in the *A. tumefaciens* WT strain and the triple SDP mutant. DUF1127 genes from *S. meliloti* (SM), *R. sphaeroides* (RSP) and *E. coli* (YjiS^EC^) were expressed from the pSRK vector. Bacteria were cultivated in YEB medium. The stained 16S rRNA served as a loading control. The Northern blot was performed in technical duplicates. (B) qPCR analysis to confirm *pstS* transcript levels. Bacteria were cultivated in YEB medium, and the qRT-PCR was performed in technical duplicates. Standard deviations are indicated by error bars.

To assess the functional conservation of these proteins, we conducted complementation experiments in *A. tumefaciens* to determine whether heterologous DUF1127 proteins could regulate *pstS* expression. Consistent with previous RNA-sequencing and Northern blot results (Kraus et al. 2020), Northern blotting confirmed that *pstS* transcription was strongly upregulated in the *A. tumefaciens* triple SDP mutant (Fig. 6A). The same was observed for the empty vector control, indicating that the vector itself had no influence on *pstS* transcription. Complementation with a plasmid expressing *A. tumefaciens* SDP1 restored *pstS* expression to low WT-like levels. Remarkably, plasmids encoding DUF1127 proteins from *S. meliloti* (SM2011_RS33625; Hadjeras et al. 2023), which shares 72% homology with SDP1, *R. sphaeroides* (RSP_6037, CcaF1; Grützner et al. 2021), with 34% homology, and *E. coli* (YjiS; Sibley and Raleigh 2004), with 24% homology, were all able to fully complement the phenotype (Figs. S3 and 6A).

qPCR analysis further validated the Northern blot results, confirming that *pstS* transcripts were highly upregulated in the triple SDP mutant with or without the empty vector (Fig. 6B). Once again, expression of DUF1127 proteins from diverse bacterial species reduced *pstS* expression to WT levels. These findings suggest that DUF1127 proteins function through a conserved regulatory mechanism to control phosphate uptake across different bacterial taxa.

### Phosphate-related phenotypes in the *E. coli yjiS* mutant

**Figure 7.**
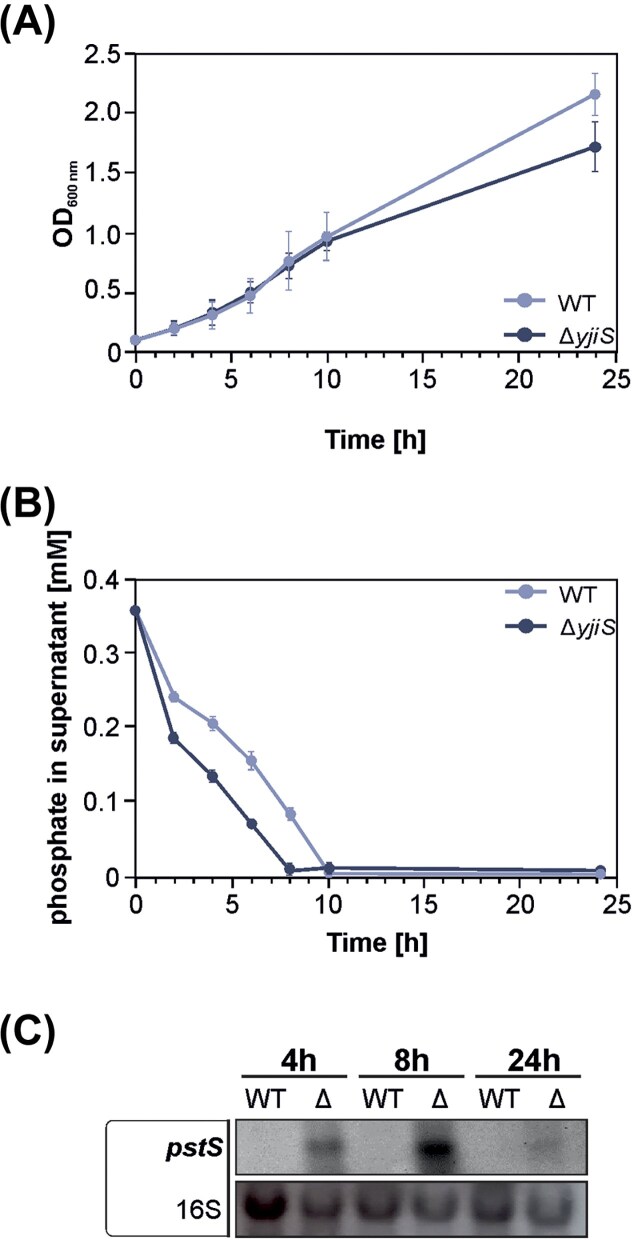
Phenotypic characterization of the *E. coli yjiS* mutant with respect to phosphate metabolism. (A) Growth curves of the *E. coli* WT strain and the *yjiS* mutant in MM with trehalose. The experiment was performed in biological triplicates. Standard deviations are indicated by error bars. (B) Phosphate levels remaining in the supernatant of the WT strain and the *yjiS* mutant. Experiment was performed in biological duplicates. Standard deviations are indicated by error bars. (C) Northern blot analysis of the *pstS* transcript in the WT and *yjiS* mutant. Bacteria were cultivated in MM. The experiment was performed in biological duplicates.

The results presented above suggest that the sole *E. coli* DUF1127 protein, YjiS, may share the same physiological function as the *A. tumefaciens* SDPs. To investigate this possibility, we analyzed growth, phosphate uptake and *pstS* expression in *E. coli* WT and the corresponding *yjiS* mutant. Reminiscent of the phenotypes observed in *A. tumefaciens* (Fig. 2), the *E. coli yjiS* mutant exhibited a slight growth defect in MM after 10 h and took up phosphate faster than the WT (Fig. 7A and B). Additionally, Northern blot analysis revealed that *pstS* transcript levels were upregulated in the *yjiS* mutant (Fig. 7C). These findings further support the notion that the DUF1127 proteins play a conserved role in regulating phosphate homeostasis across different bacterial species.

### Interaction between *E. coli* YjiS and PhoR

**Figure 8.**
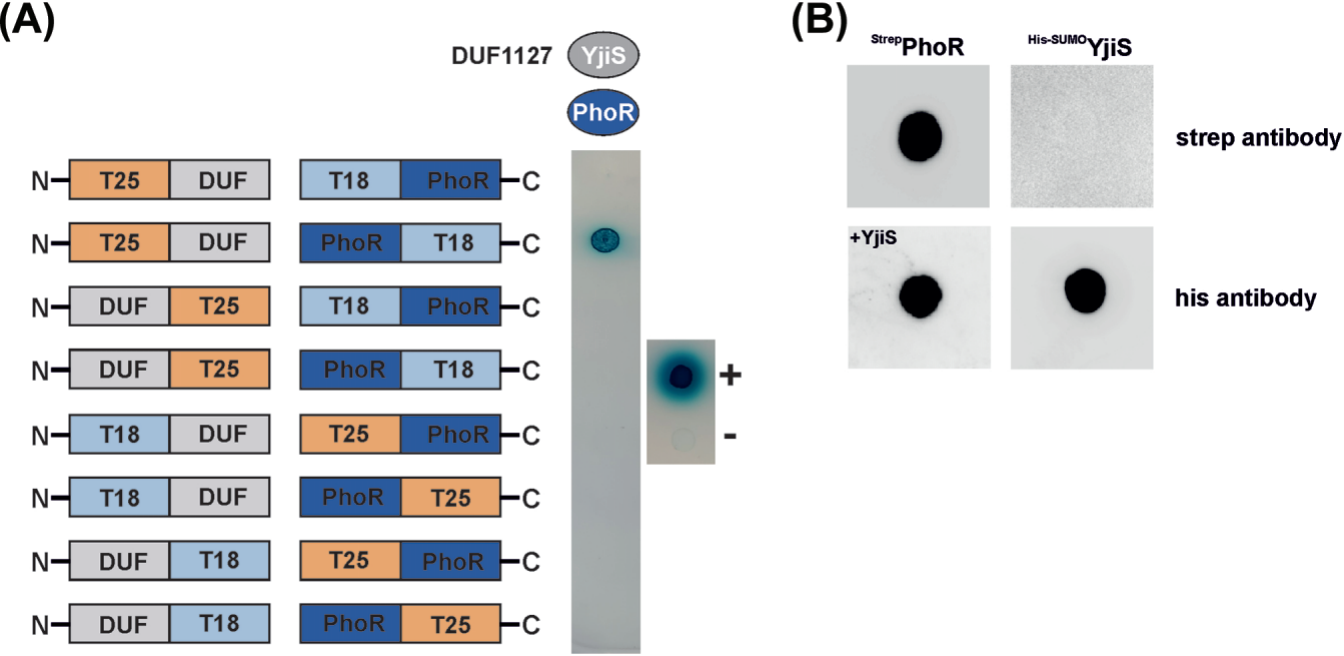
Protein–protein interaction between *E. coli* PhoR and YjiS. (A) The bacterial-two hybrid system in the adenylate cyclase-negative *E. coli* reporter strain DHM1. The positive control is spotted above the negative control (−) on the right side of both plates. (B) Far–Western dot blot with purified PhoR and YjiS from *E. coli*. The experiment was performed in biological triplicates.

Given the striking similarities in phosphate-related phenotypes between *E. coli* and *A. tumefaciens* DUF1127 mutants, we investigated whether *E. coli* YjiS interacts with the sensor kinase PhoR. Unlike the two-hybrid assay results with *Agrobacterium* SDPs, where the fusion orientation did not play a role (Fig. 5), blue colonies were observed only when YjiS was C-terminally fused to the T25 domain and PhoR was N-terminally fused to the T18 domain (Fig. 8A). This result highlights a common challenge in two-hybrid analysis, where the fused adenylate cyclase domain can obscure interactions, requiring multiple fusion constructs in different orientations (Möller et al. 2023). To further validate this putative interaction by an *in vitro* approach with purified proteins, we employed Far–Western blotting, which confirmed the direct interaction between *E. coli* YjiS and PhoR (Fig. 8B).

Cumulatively, our results suggest that DUF1127 proteins have a conserved function across bacterial species and exert their regulatory role in phosphate metabolism through direct interaction with the sensor kinase PhoR.

## Discussion

### Numerous physiological roles of DUF1127 proteins

In recent years, small proteins have attracted growing interest due to their diverse roles in bacterial physiology. Among these, proteins containing DUFs are particularly intriguing because of their widespread presence across a broad range of organisms. However, functional analysis of these proteins is often challenging, as mutants frequently display no observable phenotype, complicating efforts to determine their biological roles (Storz et al. 2014, VanOrsdel et al. 2018).

A notable exception is the family of small DUF1127-containing proteins. Deletion of all three SDPs in *A. tumefaciens* resulted in substantial transcriptomic reprogramming, accompanied by a wide range of phenotypic changes (Kraus et al. 2020), indicating a significant physiological impact of these proteins (Alakavuklar and Fuqua 2020). The phenotypic alterations in the mutant included enhanced cell aggregation, increased biofilm formation, and modifications in exopolysaccharide production as well as changes in carbon and nitrogen utilization. Phenotypes with respect to carbon metabolism have also been observed in *B. abortus* and *R. sphaeroides* (Billenkamp et al. 2015, Budnick et al. 2018) and with respect to biofilm formation in *V. alginolyticus* (Feng et al. 2024). In *Salmonella*, the DUF1127 protein YjiS was produced under infection-mimicking conditions and delayed the escape from infected macrophages (Venturini et al. 2025). Cumulatively, these results emphasize a broad, yet largely unexplored physiological role of DUF1127 proteins.

One of the most striking findings in the *A. tumefaciens* triple SDP mutant was the pronounced upregulation of phosphate acquisition genes, observed at both the transcriptomic and proteomic level (Kraus et al. 2020). The concurrent induction of three distinct uptake systems for phosphate or other phosphorus-containing compounds suggests a coordinated regulatory mechanism, likely mediated by the PhoRB two-component system (Fig. 1).

### How do small DUF1127 proteins coordinate phosphate uptake?

Given the indispensable functions of phosphate in cellular metabolism and the highly variable concentrations of this nutrient in the environment, bacteria must be able to sense extracellular phosphate levels and tightly regulate its uptake and intracellular distribution. In this study, we found that the upregulation of phosphate uptake systems, particularly the high-affinity Pst system, in the *Agrobacterium* triple SDP mutant led to accelerated phosphate import. This, in turn, resulted in premature growth arrest and intracellular accumulation of polyphosphate.

The canonical bacterial phosphate signaling pathway, extensively characterized in *E. coli* and also present in *A. tumefaciens*, consists of the PhoR-PhoB two-component system (Makino et al. 1989, Danhorn et al. 2004, Lamarche et al. 2008) Under phosphate-limited conditions, the sensor kinase PhoR phosphorylates PhoB, which subsequently activates transcription of the *pst* locus (Fig. 1) along with other members of the Pho regulon (Gardner and McCleary 2019).

PhoR is a homodimeric, bifunctional histidine autokinase and phospho-PhoB phosphatase. Its architecture comprises a membrane-spanning region, a cytoplasmic charged region (CR) followed by a Per-ARNT-Sim (PAS) domain and a prototypical histidine kinase domain (Gardner and McCleary 2019). When phosphate is limiting, PhoR autophosphorylates a conserved histidine residue and transfers the phosphoryl group to the response regulator PhoB. In phosphate-replete conditions, PhoR dephosphorylates PhoB (Makino et al. 1989, Carmany et al. 2003).

Unlike typical membrane-located sensor kinases, PhoR lacks a periplasmic sensory domain and is therefore unable to directly sense extracellular inorganic phosphate (Pi) levels. In *E. coli*, PhoR indirectly interacts with the high-affinity phosphate transporter PstSCAB via the connector protein PhoU. It has been proposed that PhoR switches between kinase and phosphatase activities by detecting the transporter’s conformational states through PhoU (Gardner et al. 2014, Vuppada et al. 2018). PhoU is a peripheral membrane protein that interacts directly with the PAS domain of the sensor kinase PhoR and with PstB, the cytoplasmic ATPase domain of the Pst ABC transporter. In the presence of sufficient phosphate, PhoU essentially acts as a brake preventing further uptake (Gardner et al. 2014, Baek and Lee 2024). In a *ΔphoU* strain, increased expression of the *phoB* regulon, polyphosphate accumulation, and growth impairment have been observed in *E. coli* (Rice et al. 2009). Some but not all the phenotypic characteristics have been reported in other bacterial species as well (Lubin et al. 2015, de Almeida et al. 2015). While PhoU is undoubtedly a regulator of Pi homeostasis, its mechanism of regulation appears to be divergent ranging from inhibiting the kinase activity of PhoR to modulating polyphosphate accumulation in different bacterial species (Baek and Lee 2024).

Interestingly, the *A. tumefaciens* triple SDP mutant exhibited phenotypic features reminiscent of an *E. coli ΔphoU* strain, despite having elevated *phoU* expression. This suggests the necessity of the DUF1127 proteins for PhoU-mediated PhoR kinase inhibition. Alternatively, the SDPs may inhibit PhoR in place of PhoU, while PhoU has evolved to regulate other aspects of phosphate homeostasis in *A. tumefaciens*. Considering the stark differences between the SDPs and PhoU in sequence and structure, the former hypothesis is more plausible.

**Figure 9.**
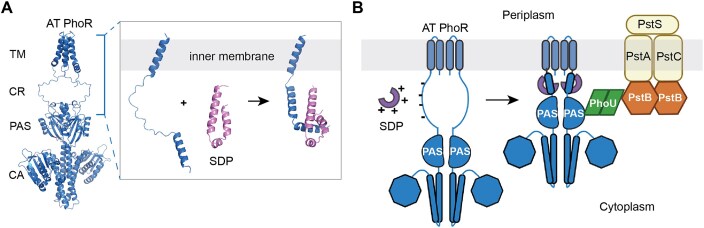
Proposed model of phosphate uptake regulation by small DUF1127 proteins in *A. tumefaciens*. (A) The PhoR dimer consists of a transmembrane domain (TM), a cytoplasmic charged region (CR), a PAS domain, and catalytic ATP-binding domains (CA). The zoomed-in view shows the CR region with flanking helices, the structure of SDP1, and the complex formed by these two molecules. All structure predictions were performed with AlphaFold3 (Abramson et al. 2024). Monomeric forms of the CR region and SDP were used for simplicity. (B) The positively charged SDP interacts with the negatively charged CR region in PhoR, inducing a conformational change. This allows the proper interaction between the PAS domain and PhoU, and a regulated phosphate uptake.

A sequence comparison of *A. tumefaciens* PhoR, PhoU, SDP, and PstB with previously studied homologs in other species indicates that the phosphate import regulatory system from *S. meliloti* is the most closely related. *Sinorhizobium meliloti* PhoU interacts with PhoR and PstB, reducing Pi import at high phosphate conditions (DiCenzo et al. 2017). Structural predictions of AT PhoR and SM PhoR reveal that the negatively charged CR region adopts a random loop conformation (Fig. 9A). This region becomes more alpha-helical when a positively charged SDP is added *in silico*. Since the CR region connects the PAS domain to the transmembrane helix in PhoR, its conformation potentially determines the distance and orientation of the PAS domain relative to the inner membrane. Therefore, we hypothesize that the SDPs regulate the phosphate import system by binding to the CR region of PhoR, causing a conformational change that positions the PAS domain properly to interact with the membrane peripheral protein PhoU (Fig. 9B). The interaction switches PhoR to its phosphatase activity and dephosphorylates PhoB, leading to a rapid shutdown of the phosphate import system. Without SDPs, PhoU may not be able to interact with PhoR properly, resulting in uncontrolled phosphate import. Further experimentation is needed to verify this hypothesis.

### Is DUF1127 an RNA- or protein-binding domain?

A hallmark of DUF1127 proteins is their unusually high content of arginine residues. On average, they contain more than twice as many arginines (14%) as the standard protein (5.5%). Arginine is the most basic of the 20 proteinogenic amino acids and arginine-rich proteins play an important role in binding negatively charged molecules. They enhance protein–protein interactions and frequently form hydrogen bonds with the phosphate backbone of nucleic acids (Chandana and Venkatesh 2016). In eukaryotes, arginine-rich proteins are crucial for chromatin organization and play essential roles in mRNA maturation (Godin and Varani 2007).

Given the well-established involvement of arginine-rich proteins in RNA binding, it is perhaps unsurprising that CcaF1, a DUF1127 protein of 71 amino acids from *R. sphaeroides*, was shown to interact with RNA (Grützner et al. 2021, 2023). CcaF1 participates in the maturation of a cluster of four homologous small RNAs encoded directly downstream of *ccaF1* in the same operon (Billenkamp et al. 2015). Furthermore, co-immunoprecipitation experiments identified numerous CcaF1-associated mRNAs involved in diverse cellular functions, and CcaF1 was found to influence the stability of some of these transcripts (Grützner et al. 2021).

In contrast to the demonstrated RNA-binding activity of CcaF1 and likely that of two additional *Rhodobacter* DUF1127 proteins (Grützner et al. 2021, Grützner et al. 2023), there is no evidence supporting RNA interaction by the *Salmonella* DUF1127 protein YjiS (Venturini et al. 2025). YjiS was found to localize to the cytoplasmic membrane, and its putative interaction partners were predominantly inner membrane proteins, suggesting that these interactions are direct, rather than RNA-mediated. Notably, one of the interaction partners of YjiS was the sensor kinase SsrA of the SsrA–SsrB two-component system. This regulatory system controls genes within the *Salmonella* pathogenicity island 2, which encodes the type III secretion system essential for intracellular survival (Cirillo et al. 1998, Pérez-Morales et al. 2017). In addition, SsrA–SsrB modulates biofilm formation and motility regulons, and depletion of *Salmonella yjiS* led to upregulation of flagellar gene expression and an increased number of flagella per cell (Venturini et al. 2025).

The ability of DUF1127 proteins to bind sensor kinases, as demonstrated for *Salmonella* YjiS, is further supported by our findings showing that the three *A. tumefaciens* SDPs interact with the sensor kinase PhoR. Several lines of evidence suggest that this interaction may be conserved across distantly related bacterial species. Complementation of the *A. tumefaciens* triple SDP mutant with DUF1127 proteins from *Sinorhizobium, Rhodobacter* (both alphaproteobacteria), or even the gammaproteobacterium *E. coli* successfully restored *pstS* transcript levels to those observed in the WT. Furthermore, both *in vivo* and *in vitro* assays indicated that *E. coli* YjiS interacts with its endogenous PhoR protein, strengthening the hypothesis that DUF1127–PhoR interactions represent a conserved mechanism for regulating phosphate homeostasis. Uncovering the precise mechanistic details of this process will be a compelling objective for future research.

Our findings are consistent with previous reports showing that small proteins frequently interact with and modulate the activity of sensor kinases (Yadavalli and Yuan 2022). A well-characterized example is the small protein MgrB in *E. coli*, which consists of 47 amino acids. MgrB inhibits the PhoP/PhoQ two-component system by binding to the periplasmic domain of the sensor kinase PhoQ. Normally, PhoQ is activated through autophosphorylation and subsequently transfers the phosphate group to the response regulator PhoP. This signaling pathway is redox-sensitive, with PhoP-dependent transcription being activated under reducing conditions in the periplasm. Localized to the inner membrane, MgrB suppresses this pathway by binding to PhoQ, thereby inhibiting its kinase activity and facilitating dephosphorylation of PhoP (Yuan et al. 2017, Yadavalli et al. 2020). Interestingly, PhoQ is also regulated by another small protein, SafA, which is 65 amino acids long. In contrast to MgrB, which serves as an inhibitor, SafA acts as an activator. Its C-terminal periplasmic region interacts with the sensor domain of PhoQ, promoting PhoQ autophosphorylation and activating downstream signaling (Ishii et al. 2013, Yoshitani et al. 2019).

The seemingly contradictory roles of DUF1127 proteins in RNA binding and protein–protein interaction remain an intriguing puzzle. This is particularly evident in the case of *Rhodobacter* CcaF1, a DUF1127 protein with documented RNA-binding activity, which nonetheless can functionally complement the phosphate defect in the *Agrobacterium* SDP mutant—a process that is mediated through protein–protein interactions. Currently, no experimental structure of a DUF1127 protein is available. However, computational predictions suggest that the abundant positively charged arginine residues are arranged in two helical structures and surface-exposed (Fig. 9). This structural organization could facilitate interactions with a wide range of negatively charged cellular components, including RNA and certain proteins. It is plausible that DUF1127 proteins function as “moonlighting proteins”—a term used to describe proteins that perform multiple, mechanistically distinct biological roles beyond their canonical function (Huberts and van der Klei 2010, Jeffery 2018). A well-known example is elongation factor Tu, which not only delivers aminoacyl–tRNA during translation but also participates in the regulation of transcription and translation through interactions with both RNA and proteins (Widjaja et al. 2017, Harvey et al. 2019).

Whether DUF1127 proteins exhibit a similar degree of functional versatility remains an open question. However, the diverse phenotypes observed upon their deletion strongly suggest that these small proteins play multiple, context-dependent roles within the cell. Future research should prioritize the identification of additional interaction partners and aim to uncover the molecular mechanisms by which DUF1127 proteins exert their regulatory functions.

## Supplementary Material

uqaf023_Supplemental_File

## Data Availability

RNA-sequencing data are available at the Gene Expression Omnibus (GEO) database under accession number GSE150941.

## References

[bib1] Abramson J, Adler J, Dunger J et al. Accurate structure prediction of biomolecular interactions with AlphaFold 3. Nature. 2024;630:493–500. 10.1038/s41586-024-07487-w. 38718835 PMC11168924

[bib2] Achbergerová L, Nahálka J. Polyphosphate–an ancient energy source and active metabolic regulator. Microb Cell Fact. 2011;10:63. 10.1186/1475-2859-10-63. 21816086 PMC3163519

[bib3] Alakavuklar MA, Fuqua C. Short, rich, and powerful: a new family of arginine-rich small proteins have outsized impact in *Agrobacterium tumefaciens*. J Bacteriol. 2020;202:e00450–20. 10.1128/JB.00450-20. 32839178 PMC7585062

[bib4] Anschutz P, Deborde J. Spectrophotometric determination of phosphate in matrices from sequential leaching of sediments. Limnol Oceanogr Meth. 2016;14:245–56. 10.1002/lom3.10085.

[bib5] Bachhawat P, Swapna GV, Montelione GT et al. Mechanism of activation for transcription factor PhoB suggested by different modes of dimerization in the inactive and active states. Structure. 2005;13:1353–63. 10.1016/j.str.2005.06.006. 16154092 PMC3685586

[bib6] Baek S, Lee EJ. PhoU: a multifaceted regulator in microbial signaling and homeostasis. Curr Opin Microbiol. 2024;77:102401. 10.1016/j.mib.2023.102401. 37988810

[bib7] Billenkamp F, Peng T, Berghoff BA et al. A cluster of four homologous small RNAs modulates C1 metabolism and the pyruvate dehydrogenase complex in *Rhodobacter sphaeroides* under various stress conditions. J Bacteriol. 2015;197:1839–52. 10.1128/JB.02475-14. 25777678 PMC4402390

[bib8] Budnick JA, Sheehan LM, Kang L et al. Characterization of three small proteins in *Brucella abortus* linked to fucose utilization. J Bacteriol. 2018;200:e00127–18. 10.1128/JB.00127-18. 29967118 PMC6112010

[bib9] Burton AT, Zeinert R, Storz G. Large roles of small proteins. Annu Rev Microbiol. 2024;78:1–22. 10.1146/annurev-micro-112723-083001. 38772630 PMC12005717

[bib10] Carmany DO, Hollingsworth K, McCleary WR. Genetic and biochemical studies of phosphatase activity of PhoR. J Bacteriol. 2003;185:1112–5. 10.1128/JB.185.3.1112-1115.2003. 12533489 PMC142828

[bib11] Chandana T, Venkatesh YP. Occurrence, functions and biological significance of arginine-rich proteins. Curr Protein Pept Sci. 2016;17:507–16. 10.2174/1389203717666151201192348. 26916156

[bib12] Cirillo DM, Valdivia RH, Monack DM et al. Macrophage-dependent induction of the *Salmonella* pathogenicity island 2 type III secretion system and its role in intracellular survival. Mol Microbiol. 1998;30:175–88. 10.1046/j.1365-2958.1998.01048.x. 9786194

[bib13] Danhorn T, Hentzer M, Givskov M et al. Phosphorus limitation enhances biofilm formation of the plant pathogen *Agrobacterium tumefaciens* through the PhoR-PhoB regulatory system. J Bacteriol. 2004;186:4492–501. 10.1128/JB.186.14.4492-4501.2004. 15231781 PMC438617

[bib14] de Almeida LG, Ortiz JH, Schneider RP et al. *phoU* inactivation in *Pseudomonas aeruginosa* enhances accumulation of ppGpp and polyphosphate. Appl Environ Microb. 2015;81:3006–15. 10.1128/AEM.04168-14. PMC4393453 25710363

[bib15] diCenzo GC, Sharthiya H, Nanda A et al. PhoU allows rapid adaptation to high phosphate concentrations by modulating PstSCAB transport rate in *S inorhizobium meliloti*. J Bacteriol. 2017;199:e00143–17. 10.1128/JB.00143-17. 28416708 PMC5573078

[bib16] Feng R, Chen Y, Chen T. DUF1127-containing protein and ProQ had opposite effects on biofilm formation in *Vibrio alginolyticus*. BMC Microbiol. 2024;24:330 10.1186/s12866-024-03486-z. 39244528 PMC11380419

[bib17] Gardner SG, Johns KD, Tanner R et al. The PhoU protein from *Escherichia coli* interacts with PhoR, PstB, and metals to form a phosphate-signaling complex at the membrane. J Bacteriol. 2014;196:1741–52. 10.1128/JB.00029-14. 24563032 PMC3993317

[bib18] Gardner SG, McCleary WR. Control of the *phoBR* regulon in *Escherichia coli*. EcoSal Plus. 2019;8:1–20. 10.1128/ecosalplus.ESP-0006-2019. PMC11573284 31520469

[bib19] Gelvin SB . *Agrobacterium*-mediated plant transformation: the biology behind the “gene-jockeying” tool. Microbiol Molecul Biol Rev. 2003;67:16–37. 10.1128/mmbr.67.1.16-37.2003. PMC150518 12626681

[bib20] Godin KS, Varani G. How arginine-rich domains coordinate mRNA maturation events. RNA Biol. 2007;4:69–75. 10.4161/rna.4.2.4869. 17873524

[bib21] Goodacre NF, Gerloff DL, Uetz P. Protein domains of unknown function are essential in bacteria. mBio. 2013;5:e00744–13. 10.1128/mBio.00744-13. 24381303 PMC3884060

[bib22] Gray MJ, Jakob U. Oxidative stress protection by polyphosphate–new roles for an old player. Curr Opin Microbiol. 2015;24:1–6. 10.1016/j.mib.2014.12.004. 25589044 PMC4380828

[bib23] Gray MJ, Wholey WY, Wagner NO et al. Polyphosphate is a primordial chaperone. Mol Cell. 2014;53:689–99. 10.1016/j.molcel.2014.01.012. 24560923 PMC3996911

[bib24] Grützner J, Billenkamp F, Spanka DT et al. The small DUF1127 protein CcaF1 from *Rhodobacter sphaeroides* is an RNA-binding protein involved in sRNA maturation and RNA turnover. Nucleic Acids Res. 2021;49:3003–19. 10.1093/nar/gkab146. 33706375 PMC8034643

[bib25] Grützner J, Börner J, Jäger A et al. The small RNA-binding protein CcaF1 promotes formation of photosynthetic complexes in *Rhodobacter sphaeroides*. Int J Mol Sci. 2023;24:9515. 10.3390/ijms24119515. 37298460 PMC10253847

[bib26] Guan J, Jakob U. The protein scaffolding functions of polyphosphate. J Mol Biol. 2024;436:168504. 10.1016/j.jmb.2024.168504. 38423453 PMC11921889

[bib27] Hadjeras L, Heiniger B, Maaß S et al. Unraveling the small proteome of the plant symbiont *Sinorhizobium meliloti* by ribosome profiling and proteogenomics. Microlife. 2023;4:uqad012. 10.1093/femsml/uqad012. 37223733 PMC10117765

[bib28] Hahnfeld JM, Schwengers O, Jelonek L et al. sORFdb—a database for sORFs, small proteins, and small protein families in bacteria. BMC Genomics. 2025;26:110. 10.1186/s12864-025-11301-w. 39910485 PMC11796252

[bib29] Harvey KL, Jarocki VM, Charles IG et al. The diverse functional roles of elongation factor Tu (EF-Tu) in microbial pathogenesis. Front Microbiol. 2019;10:2351. 10.3389/fmicb.2019.02351. 31708880 PMC6822514

[bib30] Higo N, Oishi T, Yamashita A et al. Northern blot and in situ hybridization analyses of MARCKS mRNA expression in the cerebral cortex of the macaque monkey. Cereb Cortex. 2002;12:552–64. 10.1093/cercor/12.5.552. 11950773

[bib31] Hsieh YJ, Wanner BL. Global regulation by the seven-component pi signaling system. Curr Opin Microbiol. 2010;13:198–203. 10.1016/j.mib.2010.01.014. 20171928 PMC2847643

[bib32] Huberts DH, van der Klei IJ. Moonlighting proteins: an intriguing mode of multitasking. Biochim Biophys Acta. 2010;1803:520–5. 10.1016/j.bbamcr.2010.01.022. 20144902

[bib33] Ishii E, Eguchi Y, Utsumi R. Mechanism of activation of PhoQ/PhoP two-component signal transduction by SafA, an auxiliary protein of PhoQ histidine kinase in *Escherichia coli*. Biosci Biotechnol Biochem. 2013;77:814–9. 10.1271/bbb.120970. 23563556

[bib34] Jeffery CJ . Protein moonlighting: what is it, and why is it important?. Philos Trans R Soc Lond B Biol Sci. 2018;373:20160523. 10.1098/rstb.2016.0523. 29203708 PMC5717523

[bib35] Jordan B, Weidenbach K, Schmitz RA. The power of the small: the underestimated role of small proteins in bacterial and archaeal physiology. Curr Opin Microbiol. 2023;76:102384. 10.1016/j.mib.2023.102384. 37776678

[bib36] Karimova G, Dautin N, Ladant D. Interaction network among *Escherichia coli* membrane proteins involved in cell division as revealed by bacterial two-hybrid analysis. J Bacteriol. 2005;187:2233–43. 10.1128/JB.187.7.2233-2243.2005. 15774864 PMC1065216

[bib37] Kraus A, Weskamp M, Zierles J et al. Arginine-rich small proteins with a domain of unknown function, DUF1127, play a role in phosphate and carbon metabolism of *Agrobacterium tumefaciens*. J Bacteriol. 2020;202:e00309–20. 10.1128/JB.00309-20. 33093235 PMC7585064

[bib38] Lamarche MG, Wanner BL, Crépin S et al. The phosphate regulon and bacterial virulence: a regulatory network connecting phosphate homeostasis and pathogenesis. FEMS Microbiol Rev. 2008;32:461–73. 10.1111/j.1574-6976.2008.00101.x. 18248418

[bib39] Lubin EA, Henry JT, Fiebig A et al. Identification of the PhoB regulon and role of PhoU in the phosphate starvation response of *Caulobacter crescentus*. J Bacteriol. 2015;198:187–200. 10.1128/JB.00658-15. 26483520 PMC4686198

[bib40] Makino K, Shinagawa H, Amemura M et al. Signal transduction in the phosphate regulon of *Escherichia coli* involves phosphotransfer between PhoR and PhoB proteins. J Mol Biol. 1989;210:551–9. 10.1016/0022-2836(89)90131-9. 2693738

[bib41] McInerney P, Mizutani T, Shiba T. Inorganic polyphosphate interacts with ribosomes and promotes translation fidelity in vitro and in vivo. Mol Microbiol. 2006;60:438–47. 10.1111/j.1365-2958.2006.05103.x. 16573692

[bib42] Möller AM, Brückner S, Tilg LJ et al. LapB (YciM) orchestrates protein-protein interactions at the interface of lipopolysaccharide and phospholipid biosynthesis. Mol Microbiol. 2023;119:29–43. 10.1111/mmi.15005. 36464488

[bib43] Mudgal R, Sandhya S, Chandra N et al. De-DUFing the DUFs: deciphering distant evolutionary relationships of domains of unknown function using sensitive homology detection methods. Biol Direct. 2015;10:38. 10.1186/s13062-015-0069-2. 26228684 PMC4520260

[bib44] Murphy J, Riley JP. A modifed single solution for the determination of phosphate in natural waters. Anal Chem Acta. 1962;27:31–36. 10.1016/S0003-2670(00)88444-5.

[bib45] Pérez-Morales D, Banda MM, Chau NYE et al. The transcriptional regulator SsrB is involved in a molecular switch controlling virulence lifestyles of *Salmonella*. PLoS Pathog. 2017;13:e1006497. 10.1371/journal.ppat.1006497. 28704543 PMC5562331

[bib46] Price MN, Wetmore KM, Waters RJ et al. Mutant phenotypes for thousands of bacterial genes of unknown function. Nature. 2018;557:503–9. 10.1038/s41586-018-0124-0. 29769716

[bib47] Rice CD, Pollard JE, Lewis ZT et al. Employment of a promoter-swapping technique shows that PhoU modulates the activity of the PstSCAB2 ABC transporter in *Escherichia coli*. Appl Environ Microb. 2009;75:573–82. 10.1128/AEM.01046-08. PMC2632118 19047379

[bib48] Santos-Beneit F . The pho regulon: a huge regulatory network in bacteria. Front Microbiol. 2015;6:402. 10.3389/fmicb.2015.00402. 25983732 PMC4415409

[bib49] Schmidt JJ, Remme DCLE, Eisfeld J et al. The LysR-type transcription factor LsrB regulates beta-lactam resistance in *Agrobacterium tumefaciens*. Mol Microbiol. 2024;121:26–39. 10.1111/mmi.15191. 37985428

[bib50] Schweizer H, Argast M, Boos W. Characteristics of a binding protein-dependent transport system for sn-glycerol-3-phosphate in *Escherichia coli* that is part of the pho regulon. J Bacteriol. 1982;150:1154–63. 10.1128/jb.150.3.1154-1163.1982. 7042685 PMC216336

[bib51] Sibley MH, Raleigh EA. Cassette-like variation of restriction enzyme genes in *Escherichia coli* C and relatives. Nucleic Acids Res. 2004;32:522–34. 10.1093/nar/gkh194. 14744977 PMC373321

[bib52] Stasi R, Neves HI, Spira B. Phosphate uptake by the phosphonate transport system PhnCDE. BMC Microbiol. 2019;19:79. 10.1186/s12866-019-1445-3. 30991951 PMC6469041

[bib53] Storz G, Wolf YI, Ramamurthi KS. Small proteins can no longer be ignored. Annu Rev Biochem. 2014;83:753–77. 10.1146/annurev-biochem-070611-102400. 24606146 PMC4166647

[bib54] UniProt Consortium . UniProt: the Universal protein knowledgebase. Nucleic Acids Res. 2004;32:D115–9. 10.1093/nar/gkh131. 14681372 PMC308865

[bib55] VanOrsdel CE, Kelly JP, Burke BN et al. Identifying new small proteins in *Escherichia coli*. Proteomics. 2018;18:e1700064. 10.1002/pmic.201700064. 29645342 PMC6001520

[bib56] Venturini E, Maaß S, Bischler T et al. Functional characterization of the DUF1127-containing small protein YjiS of *Salmonella typhimurium*. Microlife. 2025;6:uqae026. 10.1093/femsml/uqae026. 39790481 PMC11707872

[bib57] Vuppada RK, Hansen CR, Strickland KAP et al. Phosphate signaling through alternate conformations of the PstSCAB phosphate transporter. BMC Microbiol. 2018;18:8. 10.1186/s12866-017-1126-z. 29351743 PMC5775613

[bib58] Westermann AJ, Förstner KU, Amman F et al. Dual RNA-seq unveils noncoding RNA functions in host-pathogen interactions. Nature. 2016;529:496–501. 10.1038/nature16547. 26789254

[bib59] Widjaja M, Harvey KL, Hagemann L et al. Elongation factor Tu is a multifunctional and processed moonlighting protein. Sci Rep. 2017;7:11227. 10.1038/s41598-017-10644-z. 28894125 PMC5593925

[bib60] Wilms I, Voss B, Hess WR et al. Small RNA-mediated control of the *Agrobacterium tumefaciens* GABA binding protein. Mol Microbiol. 2011;80:492–506. 10.1111/j.1365-2958.2011.07589.x. 21320185

[bib61] Xu J, Kim J, Danhorn T et al. Phosphorus limitation increases attachment in *Agrobacterium tumefaciens* and reveals a conditional functional redundancy in adhesin biosynthesis. Res Microbiol. 2012;163:674–84. 10.1016/j.resmic.2012.10.013. 23103488 PMC3656598

[bib62] Yadavalli SS, Goh T, Carey JN et al. Functional determinants of a small protein controlling a broadly conserved bacterial sensor kinase. J Bacteriol. 2020;202:e00305–20. 10.1128/JB.00305-20. 32482726 PMC8404706

[bib63] Yadavalli SS, Yuan J. Bacterial small membrane proteins: the Swiss Army knife of regulators at the lipid bilayer. J Bacteriol. 2022;204:e0034421. 10.1128/JB.00344-21. 34516282 PMC8765417

[bib64] Yoshitani K, Ishii E, Taniguchi K et al. Identification of an internal cavity in the PhoQ sensor domain for PhoQ activity and SafA-mediated control. Biosci Biotechnol Biochem. 2019;83:684–94. 10.1080/09168451.2018.1562879. 30632929

[bib65] Yuan J, Jin F, Glatter T et al. Osmosensing by the bacterial PhoQ/PhoP two-component system. Proc Natl Acad Sci USA. 2017;114:E10792–8. 10.1073/pnas.1717272114. 29183977 PMC5740661

