# Supplementary material for: Small DUF1127 proteins regulate bacterial phosphate metabolism through protein–protein interactions with the sensor kinase PhoR
Source: Microlife. 2025 Sep 18;6:uqaf023. doi: 10.1093/femsml/uqaf023 (PMC12501420; doi:10.1093/femsml/uqaf023)
Supplement: uqaf023_Supplemental_File [file uqaf023_supplemental_file.pdf]

Supplementary information

The small DUF1127 proteins regulate bacterial phosphate metabolism through protein-protein interactions with the sensor kinase PhoR

Donata C. L. E. Remme[1], Lea-Janina Tilg[1], Yvonne Pfänder[1], Jing Yuan[2], and Franz Narberhaus[1#]

[1] Microbial Biology, Ruhr University Bochum, Bochum, Germany

[2] Max Planck Institute for Terrestrial Microbiology and Center for Synthetic Microbiology, Marburg, Germany

[#]For correspondence. E-mail franz.narberhaus@rub.de

https://orcid.org/0000-0002-8552-5310

Supplementary information:

Figure S1. *In vitro* interaction studies with PstS, PstB, PhoU, PhoB and SDP3

Figure S2. Pulldown experiments showing interaction between SDP2/3 and PhoR.

Figure S3. Amino acid sequence comparison of DUF1127 proteins

Table S1. Bacterial strains used in this study.

Table S2. Plasmids used in this study.

Table S3. Oligonucleotides used in this study.

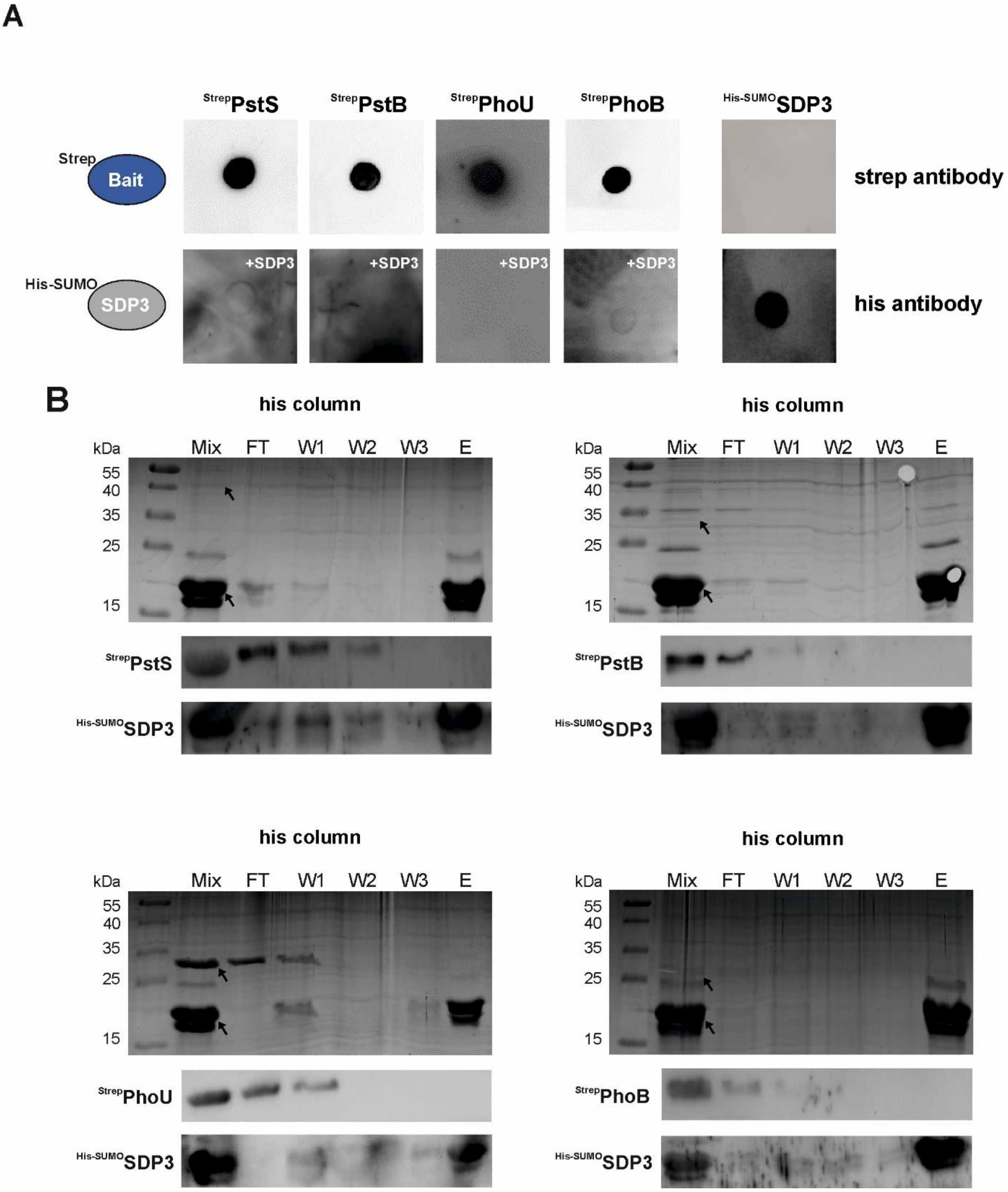

Figure. S1. *In vitro* interaction studies with PstS, PstB, PhoU, PhoB and SDP3 (A) Far-Western dot blot analysis was performed using purified SDP3 and purified PstS, PstB, PhoU and PhoB from *A. tumefaciens*. Strep tagged proteins were spotted onto a nitrocellulose membrane, which was then incubated with SDP3. Bait proteins were detected by the Strep-antibody and SDP3 was detected by the His antiboby. **(B)** Pulldown assays to examine the interaction between PstS, PstB, PhoU, PhoB and SDP3 using His columns. Coomassie-stained SDS-PAGE gels are shown on top and immunoblots at the bottom. The position of bands in the Coomassie-stained gel corresponding to those detected in the Western blot are indicated by an arrow.

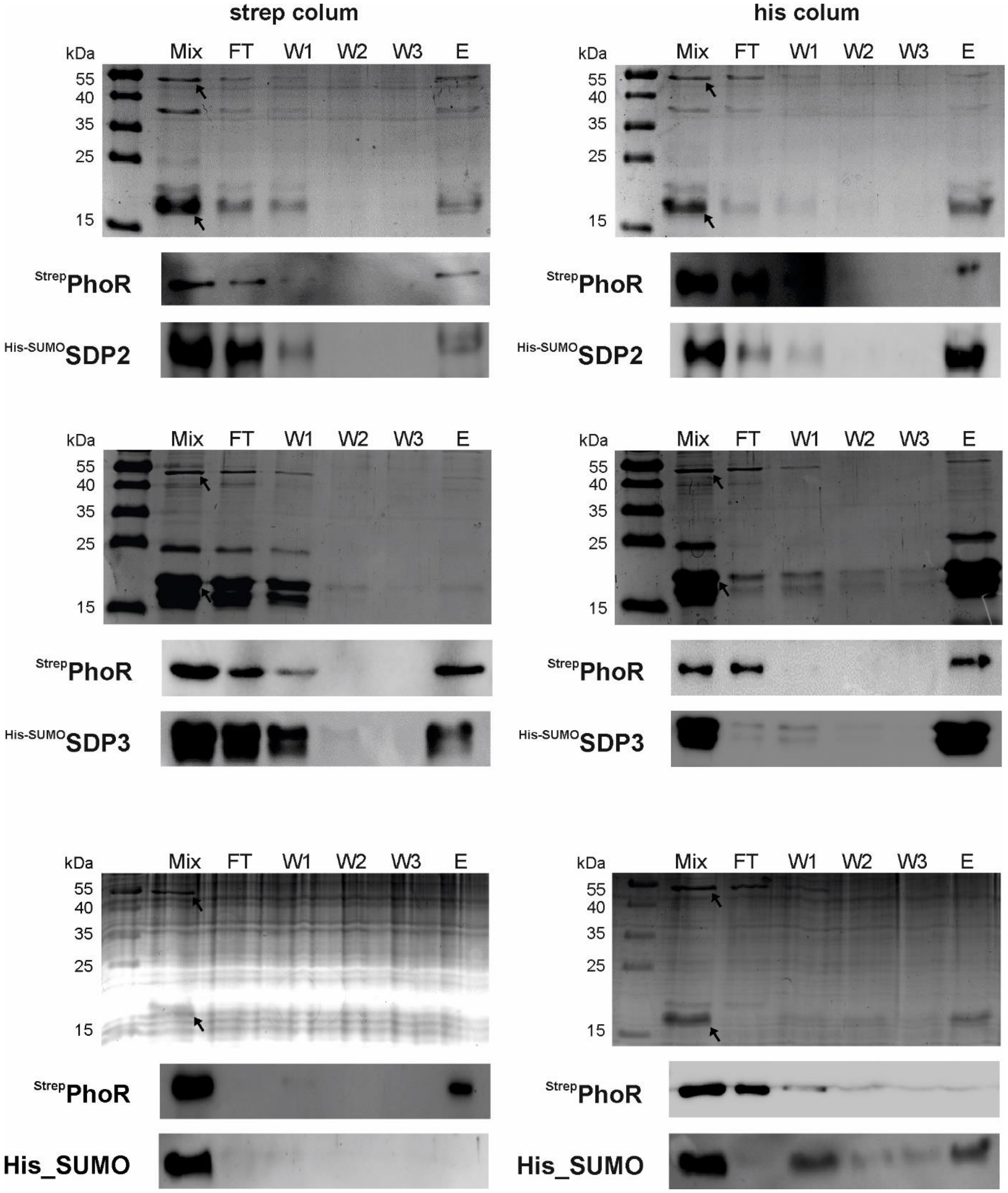

Figure. S2. **Pulldown experiments showing interaction between SDP2/3 and PhoR.** The experiment with the His-SUMO tag alone and PhoR served as a negative control. A his (right) or strep (left) columns were used. For each experiment, Coomassie-stained SDS-PAGE gels are shown on top and immunoblots at the bottom. The position of bands in the Coomassie-stained gel corresponding to those detected in the Western blot are indicated by an arrow.

A

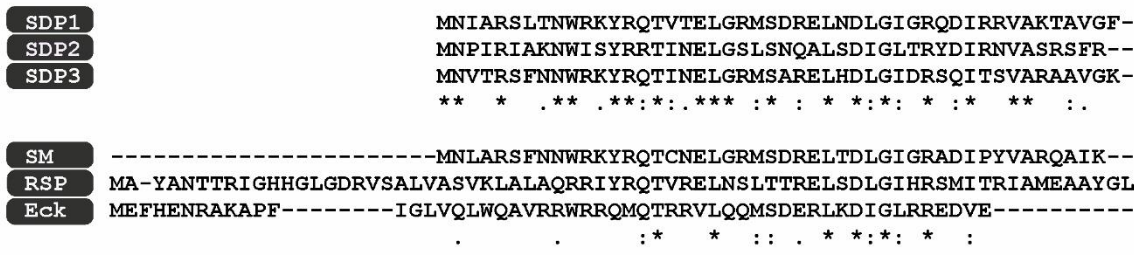

B

Organism	Protein	Name	SDP1 homology [%]
A. tumefaciens C58	Atu1667	SDP1	100%
S. meliloti 2011	SM2011_RS33625	SM	72.3%
R. sphaeroides	RSP_6037	RSP	34.3%
E. coli K-12	Eck4332	YjiS	24.1%

Figure. S3. **Amino acid sequence comparison of DUF1127 proteins. (A)** Comparison of the amino acid sequences of the three SDPs from *A. tumefaciens* and the DUF1127 proteins from *S. meliloti* (SM), *R. sphaeroides* (RSP), and *E. coli* (YjiS). * = identical; : = strong similarity; . = weak similarity **(B)** The similarity of the DUF1127 protein sequences from *S. meliloti*, *R. sphaeroides*, and *E. coli* to SDP1 was calculated.

Table S1. Bacterial strains used in this study.

Strain	Description	Source
A. tumefaciens C58	*Agrobacterium tumefaciens* wild type	C. Baron, Montreal, Canada
A. tumefaciens C58 *Δatu1667, Δatu8161, ΔL4* (ΔΔΔ)	Marker-less deletion of *L4*, *atu1667* and *atu8161*	Kraus, 2020
A. tumefaciens C58 *ΔpstS*	Marker-less deletion of *pstS* (*atu0420*) in *A. tumefaciens* C58	This study
A. tumefaciens C58 *Δatu1667, Δatu8161, ΔL4, ΔpstS* (ΔΔΔΔ)	Marker-less deletion of *pstS* (*atu0420*) in *A. tumefaciens* ΔΔΔ	This study
A. tumefaciens C58 *Δatu1667, Δatu8161, ΔL4* +pSRK	Marker-less deletion of *L4*, *atu1667* and *atu8161* with the pSRK empty vector. Kanamycin resistant.	This study
A. tumefaciens C58 *Δatu1667, Δatu8161, ΔL4* +pRNA76	Marker-less deletion of *L4*, *atu1667* and *atu8161* with pRNA76. Kanamycin resistant.	This study
A. tumefaciens C58 *Δatu1667, Δatu8161, ΔL4* +pLT14	Marker-less deletion of *L4*, *atu1667* and *atu8161* with pLT14. Kanamycin resistant.	This study
A. tumefaciens C58 *Δatu1667, Δatu8161, ΔL4* +pLT8	Marker-less deletion of *L4*, *atu1667* and *atu8161* with pLT8. Kanamycin resistant.	This study
A. tumefaciens C58 *Δatu1667, Δatu8161, ΔL4* +pLT4	Marker-less deletion of *L4*, *atu1667* and *atu8161* with pLT4. Kanamycin resistant.	This study
E. coli MG1655	*Escherichia coli* wild type	Bachmann, 1972
E. coli ΔyjiS	Marker-less deletion of *yjiS* (*eck0394*) in *E. coli* MG1655	This study
Bl21	*E. coli* strain for heterologe protein production. F-, ompT, gal(dcm)(lon), hsdSB ($rB^{-}mB^{-}$), λ DE3]	Studier & Moffatt, 1986
Bl21+pE-SUMO-Amp	*E. coli* Bl21 strain with pE-SUMO-Amp. Ampicillin resistant.	This study
Bl21+pRNA32	*E. coli* Bl21 strain with pRNA32. Ampicillin resistant.	This study
Bl21+pRNA33	*E. coli* Bl21 strain with pRNA33. Ampicillin resistant.	This study
Bl21+pRNA34	*E. coli* Bl21 strain with pRNA34. Ampicillin resistant.	This study
Bl21+pDCR27	*E. coli* Bl21 strain with pDCR27. Ampicillin resistant.	This study
Bl21+pDCR81	*E. coli* Bl21 strain with pDCR81. Ampicillin resistant.	This study
Bl21+pLT19	*E. coli* Bl21 strain with pLT19. Ampicillin resistant.	This study
Bl21+pDCR24	*E. coli* Bl21 strain with pDCR24. Ampicillin resistant.	This study
DHM1	F-, cya-854, recA1, endA1, gyrA96 (Nalr), thi1, hsdR17,spoT1, rfbD1, glnV44(AS)	Karimova et al., 1998
DHM1+pDCR59+pDCR77	*E. coli* DHM1 strain with pDCR59 and pDCR77. Ampicillin and kanamycin resistant.	This study
DHM1+pDCR59+pDCR76	*E. coli* DHM1 strain with pDCR59 and pDCR76. Ampicillin and kanamycin resistant.	This study
DHM1+pDCR60+pDCR77	*E. coli* DHM1 strain with pDCR60 and pDCR77. Ampicillin and kanamycin resistant.	This study
DHM1+pDCR60+pDCR76	*E. coli* DHM1 strain with pDCR60 and pDCR76. Ampicillin and kanamycin resistant.	This study
DHM1+pDCR62+pDCR74	*E. coli* DHM1 strain with pDCR62 and pDCR74. Ampicillin and kanamycin resistant.	This study
DHM1+pDCR62+pDCR75	*E. coli* DHM1 strain with pDCR62 and pDCR75. Ampicillin and kanamycin resistant.	This study
DHM1+pDCR61+pDCR74	*E. coli* DHM1 strain with pDCR61 and pDCR74. Ampicillin and kanamycin resistant.	This study
DHM1+pDCR61+pDCR75	*E. coli* DHM1 strain with pDCR61 and pDCR75. Ampicillin and kanamycin resistant.	This study
DHM1+pDCR63+pDCR77	*E. coli* DHM1 strain with pDCR63 and pDCR77. Ampicillin and kanamycin resistant.	This study
DHM1+pDCR63+pDCR76	*E. coli* DHM1 strain with pDCR63 and pDCR76. Ampicillin and kanamycin resistant.	This study
DHM1+pDCR64+pDCR77	*E. coli* DHM1 strain with pDCR64 and pDCR77. Ampicillin and kanamycin resistant.	This study
DHM1+pDCR64+pDCR76	*E. coli* DHM1 strain with pDCR64 and pDCR76. Ampicillin and kanamycin resistant.	This study

DHM1+pDCR66+pDCR74	*E. coli* DHM1 strain with pDCR66 and pDCR74. Ampicillin and kanamycin resistant.	This study
DHM1+pDCR66+pDCR75	*E. coli* DHM1 strain with pDCR66 and pDCR75. Ampicillin and kanamycin resistant.	This study
DHM1+pDCR65+pDCR74	*E. coli* DHM1 strain with pDCR65 and pDCR74. Ampicillin and kanamycin resistant.	This study
DHM1+pDCR65+pDCR75	*E. coli* DHM1 strain with pDCR65 and pDCR75. Ampicillin and kanamycin resistant.	This study
DHM1+pDCR67+pDCR77	*E. coli* DHM1 strain with pDCR67 and pDCR77. Ampicillin and kanamycin resistant.	This study
DHM1+pDCR67+pDCR76	*E. coli* DHM1 strain with pDCR67 and pDCR76. Ampicillin and kanamycin resistant.	This study
DHM1+pDCR68+pDCR77	*E. coli* DHM1 strain with pDCR68 and pDCR77. Ampicillin and kanamycin resistant.	This study
DHM1+pDCR68+pDCR76	*E. coli* DHM1 strain with pDCR68 and pDCR76. Ampicillin and kanamycin resistant.	This study
DHM1+pDCR70+pDCR74	*E. coli* DHM1 strain with pDCR70 and pDCR74. Ampicillin and kanamycin resistant.	This study
DHM1+pDCR70+pDCR75	*E. coli* DHM1 strain with pDCR70 and pDCR75. Ampicillin and kanamycin resistant.	This study
DHM1+pDCR69+pDCR74	*E. coli* DHM1 strain with pDCR69 and pDCR74. Ampicillin and kanamycin resistant.	This study
DHM1+pDCR69+pDCR75	*E. coli* DHM1 strain with pDCR69 and pDCR75. Ampicillin and kanamycin resistant.	This study
DHM1+pDCR66+pDCR67	*E. coli* DHM1 strain with pDCR66 and pDCR67. Ampicillin and kanamycin resistant.	This study
DHM1+pDCR65+pDCR67	*E. coli* DHM1 strain with pDCR65 and pDCR67. Ampicillin and kanamycin resistant.	This study
DHM1+pDCR66+pDCR59	*E. coli* DHM1 strain with pDCR66 and pDCR59. Ampicillin and kanamycin resistant.	This study
DHM1+pDCR65+pDCR59	*E. coli* DHM1 strain with pDCR65 and pDCR59. Ampicillin and kanamycin resistant.	This study
DHM1+pDCR62+pDCR67	*E. coli* DHM1 strain with pDCR62 and pDCR67. Ampicillin and kanamycin resistant.	This study
DHM1+pDCR61+pDCR67	*E. coli* DHM1 strain with pDCR61 and pDCR67. Ampicillin and kanamycin resistant.	This study
DHM1+pKT25-zip+pUT18C-zip	*E. coli* DHM1 strain with pKT25-zip and pUT18C-zip. Ampicillin and kanamycin resistant.	This study
DHM1+pKT25+pUT18C	*E. coli* DHM1 strain with pKT25 and pUT18-C. Ampicillin and kanamycin resistant.	This study
DHM1+pDCR89+pDCR88	*E. coli* DHM1 strain with pDCR89 and pDCR88. Ampicillin and kanamycin resistant.	This study
DHM1+pDCR89+pDCR87	*E. coli* DHM1 strain with pDCR89 and pDCR87. Ampicillin and kanamycin resistant.	This study
DHM1+pDCR90+pDCR88	*E. coli* DHM1 strain with pDCR90 and pDCR88. Ampicillin and kanamycin resistant.	This study
DHM1+pDCR90+pDCR87	*E. coli* DHM1 strain with pDCR90 and pDCR87. Ampicillin and kanamycin resistant.	This study
DHM1+pDCR92+pDCR89	*E. coli* DHM1 strain with pDCR92 and pDCR89. Ampicillin and kanamycin resistant.	This study
DHM1+pDCR92+pDCR90	*E. coli* DHM1 strain with pDCR92 and pDCR90. Ampicillin and kanamycin resistant.	This study
DHM1+pDCR91+pDCR89	*E. coli* DHM1 strain with pDCR91 and pDCR89. Ampicillin and kanamycin resistant.	This study
DHM1+pDCR91+pDCR90	*E. coli* DHM1 strain with pDCR91 and pDCR90. Ampicillin and kanamycin resistant.	This study

Table S2. Plasmids used in this study.

Plasmid	Description	Source
pk19mobsacB	Cloning vector for marker less deletion mutants.	Schäfer et al., 1994
pDCR1	For marker-less deletion of *atu0420*. PCR fragment 1 (primers 1 & 2) was digested with PstI and BamHI, and PCR fragment 2 (primers 3 & 4) with BamHI and EcoRI. The fragments were ligated into the pk19mobsacB vector, which had been digested with PstI and EcoRI.	This study
pSRK	Complementation vector. Kanamycin resistant.	Torres-Quesada et al., 2013
pRNA76	Complementation vector pSRK with SDP1 (*atu1667)*. The PCR fragment (primers 5& 6), digested with XhoI, was ligated into pSRK, digested with SalI.	This study
pLT14	Complementation vecctor pSRK with *SM2011_RS33625*. The PCR fragment (primers 7 & 8), digested with XhoI, was ligated into pSRK, digested with SalI. The fragment with the sequence listed in Table S4 was used as a template for the PCR.	This study
pLT8	Complementation vector pSRK with *RSP_RS03480*. The PCR fragment (primers 9 & 10), digested with XhoI, was ligated into pSRK, digested with SalI. The fragment with the sequence listed in Table S4 was used as a template for the PCR.	This study
pLT4	Complementation vector pSRK with *eck4332* (*yjiS*). The PCR fragment (primers 11 & 12), digested with XhoI, was ligated into pSRK, digested with SalI. The fragment with the sequence listed in Table S4 was used as a template for the PCR.	This study
pLT14	Complementation vector pSRK with *SM2011_RS33625*. The PCR fragment (primers 7 & 8), digested with XhoI, was ligated into pSRK, digested with SalI.	This study
pLT8	Complementation vector pSRK with *RSP_RS03480*. The PCR fragment (primers 9 & 10), digested with XhoI, was ligated into pSRK, digested with SalI.	This study
pLT4	Complementation vector pSRK with *eck4332* (*yjiS*). The PCR fragment (primers 11 & 12), digested with XhoI, was ligated into pSRK, digested with SalI.	This study
pE-SUMO_ Amp	For IPTG-inducible expression of the His-SUMO tag alone or fused proteins tot he His-SUMO tag in *E. coli.* Ampicillin resistant.	LifeSensor Inc.
pRNA32	For IPTG-inducible expression of the His-SUMO-SDP3 (L4) in *E. coli.* The PCR fragment (primers 13 & 14), digested with BsaI, was ligated into pE-SUMO_Amp, also digested with BsaI.	Kraus, 2019
pRNA33	For IPTG-inducible expression of the His-SUMO-SDP1 (Atu1667) in *E. coli.* The PCR fragment (primers 15 & 16), digested with BsaI, was ligated into pE-SUMO_Amp, also digested with BsaI.	Kraus, 2019
pRNA34	For IPTG-inducible expression of the His-SUMO-SDP2 (Atu8161) in *E. coli.* The PCR fragment (primers 17 & 18), digested with BsaI, was ligated into pE-SUMO_Amp, also digested with BsaI.	Kraus, 2019
pET28(a)	For IPTG-inducible expression of a protein with a C-terminal His tag in *E. coli.*	Novagen
pDCR24	For IPTG-inducible expression of *ppx* (*b2502*) with a C-terminal His tag in *E. coli.* The PCR fragment (primers 19 & 20), digested with HindIII and XhoI, was ligated into pET28(a), also digested with HindIII and XhoI.	This study
pASK-IBA45+	AHT-inducible Expression vector with a strep tag. Ampicillin resistant.	IBA GmbH
pDCR27	For AHT-inducible expression of the Strep-PhoR (Atu0419) in *E. coli.* Ampicillin resistant. The PCR fragment (primers 21 & 22), digested with HindIII and EcoR1, was ligated into pASK-IBA45+. also digested with HindIII and EcoR1.	This study
pASK-IBA5	AHT-inducible Expression vector with a strep tag. Ampicillin resistant.	IBA GmbH
pDCR81	For AHT-inducible expression of the Strep-PhoR (Eck0394) in *E. coli.* The PCR fragment (primers 23 & 24), digested with SalI and EcoR1, was ligated into pASK-IBA5. also digested with SalI and EcoR1.	This study
pLT19	For IPTG-inducible expression of the His-SUMO-YjiS (Eck4332) in *E. coli.* The PCR fragment (primers 25 & 26), digested with BsaI, was ligated into pE-SUMO_Amp, also digested with BsaI.	This study
pKT25	P15A, P_{lac}(T25-MCS). Kanamycin resistant.	Karimova et al., 1998
pKNT25	P15A, P_{lac}(MCS-T25). Kanamycin resistant.	Karimova et al., 1998
pUT18C	P15A, P_{lac}(MCS-T18). Ampicillin resistant.	Karimova et al., 1998
pUT18	P15A, P_{lac}(T18-MCS). Ampicillin resistant.	Karimova et al., 1998
pKT25-zip	P15A, P_{lac}(T25-GCN4). Kanamycin resistant.	Karimova et al., 2001

pUT18C-zip	P15A, P_{lac}(GCN4-T25). Ampicillin resistant.	Karimova et al., 2001
pDCR59	P15A, P_{lac}(T25-*sdp1*). The PCR fragment (primers 27 & 29), digested with BamHI and KpnI, was ligated into pKT25, also digested with BamHI and KpnI.	This study
pDCR60	P15A, P_{lac}(*sdp1*-T25). The PCR fragment (primers 27 & 28), digested with BamHI and KpnI, was ligated into pKNT25, also digested with BamHI and KpnI.	This study
pDCR61	P15A, P_{lac}(*sdp1*-T18). The PCR fragment (primers 27 & 28), digested with BamHI and KpnI, was ligated into pUT18C, also digested with BamHI and KpnI.	This study
pDCR62	P15A, P_{lac}(T18-*sdp1*). The PCR fragment (primers 27 & 29), digested with BamHI and KpnI, was ligated into pUT18, also digested with BamHI and KpnI.	This study
pDCR63	P15A, P_{lac}(T25-*sdp2*). The PCR fragment (primers 30 & 32), digested with BamHI and KpnI, was ligated into pKT25, also digested with BamHI and KpnI.	This study
pDCR64	P15A, P_{lac}(*sdp2*-T25). The PCR fragment (primers 30 & 31), digested with BamHI and KpnI, was ligated into pKNT25, also digested with BamHI and KpnI.	This study
pDCR65	P15A, P_{lac}(*sdp2*-T18). Ampicillin resistant. The PCR fragment (primers 30 & 31), digested with BamHI and KpnI, was ligated into pUT18C, also digested with BamHI and KpnI.	This study
pDCR66	P15A, P_{lac}(T18-*sdp2*). The PCR fragment (primers 30 & 32), digested with BamHI and KpnI, was ligated into pUT18, also digested with BamHI and KpnI.	This study
pDCR67	P15A, P_{lac}(T25-*sdp3*). The PCR fragment (primers 33 & 35), digested with BamHI and KpnI, was ligated into pKT25, also digested with BamHI and KpnI.	This study
pDCR68	P15A, P_{lac}(*sdp3*-T25). The PCR fragment (primers 33 & 34), digested with BamHI and KpnI, was ligated into pKNT25, also digested with BamHI and KpnI.	This study
pDCR70	P15A, P_{lac}(T18-*sdp3*). The PCR fragment (primers 33 & 35), digested with BamHI and KpnI, was ligated into pUT18, also digested with BamHI and KpnI.	This study
pDCR74	P15A, P_{lac}(T25-*atu0419*). The PCR fragment (primers 36 & 38), digested with BamHI and KpnI, was ligated into pKT25, also digested with BamHI and KpnI.	This study
pDCR75	P15A, P_{lac}(*atu0419*-T25). The PCR fragment (primers 36 & 37), digested with BamHI and KpnI, was ligated into pKNT25, also digested with BamHI and KpnI.	This study
pDCR76	P15A, P_{lac}(*atu0419*-T18). The PCR fragment (primers 36 & 37), digested with BamHI and KpnI, was ligated into pUT18C, also digested with BamHI and KpnI.	This study
pDCR77	P15A, P_{lac}(T18-*atu0419).* The PCR fragment (primers 36 & 38), digested with BamHI and KpnI, was ligated into pUT18, also digested with BamHI and KpnI.	This study
pDCR85	P15A, P_{lac}(T25-*eck0394*). The PCR fragment (primers 39 & 41), digested with BamHI and KpnI, was ligated into pKT25, also digested with BamHI and KpnI.	This study
pDCR86	P15A, P_{lac}(*eck03942*-T25). The PCR fragment (primers 39 & 40), digested with BamHI and KpnI, was ligated into pKNT25, also digested with BamHI and KpnI.	This study
pDCR87	P15A, P_{lac}(*eck0394*-T18). The PCR fragment (primers 39 & 40), digested with BamHI and KpnI, was ligated into pUT18C, also digested with BamHI and KpnI.	This study
pDCR88	P15A, P_{lac}(T18-*eck0394).* The PCR fragment (primers 39 & 41), digested with BamHI and KpnI, was ligated into pUT18, also digested with BamHI and KpnI.	This study
pDCR89	P15A, P_{lac}(T25-*eck4332*). The PCR fragment (primers 42 & 44), digested with BamHI and KpnI, was ligated into pKT25, also digested with BamHI and KpnI.	This study
pDCR90	P15A, P_{lac}(*eck4332*-T25). The PCR fragment (primers 42 & 43), digested with BamHI and KpnI, was ligated into pKNT25, also digested with BamHI and KpnI	This study
pDCR91	P15A, P_{lac}(*eck4332*-T18). The PCR fragment (primers 42 & 43), digested with BamHI and KpnI, was ligated into pUT18, also digested with BamHI and KpnI.	This study

pDCR92	P15A, P_{lac}(T18-*eck4332)*. The PCR fragment (primers 42 & 44), digested with BamHI and KpnI, was ligated into pUT18, also digested with BamHI and KpnI.	This study

Table S3. Oligonucleotides used in this study. Restriction sides are highlighted in thick letters.

Primer number and name	Sequence	Designated use
**1** UP-atu0420-P1	TTTT**CTGCAG**CCGTTCGAAAAAGGGCACGG	*pstS* deletion mutant
**2** LP-atu0420-B1	TTTT**GGATCC**CGTCGTGGGCTTGTTTGTG	
**3** UP-atu0420-B1	TTTT**GGATCC**GCCGGCGCTTCGATGTAATAAA	
**4** LP-atu0420-E1	TTTT**GAATCC**GGCGTGCCTCAAGAGCACGG	
**5** pSRK-1667-fw	TTTT**CTCGAG**AACCCTCTATAGCCACCAAAAGC	*sdp1* in pSRK
**6** pSRK-1667-rv	TTTT**CTCGAG**GCCTTTTTGGGGAAGTCGTCTTC	
**7** UP-SM2011-X	TTTT**CTCGAG**AACCCTCTATAGCCACCAAA	*SM2011_RS33625* in pSRK
**8** LP-SM2011-X	AAAA**CTCGAG**TTACTTGATTGCCTGAC	
**9** UP-RSP6037-X	TTTT**CTCGAG**AACCCTCTATAGCCACCAAA	*RSP_RS03480* in pSRK
**10** LP-RSP6037-X	AAAA**CTCGAG**TCAGAGACCGTAGGCGGCTTC	
**11** UP-ECK4332-X	TTTT**CTCGAG**AACCCTCTATAGCCACCAAA	*yjiS* in pSRK
**12** LP-ECK4332-X	AAAA**CTCGAG**TCACTCCACATCCTCCCTGC	
**13** L4-SUMO-fw	TTTT**GGTCTC**AAGGTATGAACGTAACACGCAGCTTCAAC	*sdp3* pE-SUMO_Amp
**14** L4-SUMO-rv	TTTT**GGTCTC**ACTAGTTACTTGCCGACGGCGGCGCGG	
**15** 1667-SUMO-fw	TTTT**GGTCTC**AAGGTATGAACATTGCACGCTCGCTGAC	*sdp1* pE-SUMO_Amp
**16** 1667-SUMO-rv	TTTT**GGTCTC**ACTAGTTAGAAGCCGACGGCGGTCTTGG	
**17** 8161-SUMO-fw	TTTT**GGTCTC**AAGGTATGAACCCTATCCGCATCGCAAAG	*sdp2* pE-SUMO_Amp
**18** 8161-SUMO-rv	TTTT**GGTCTC**ACTAGTTAGCGGAACGAACGGGAAGCAAC	
**19** UP-ppx-H1	TTTT**AAGCTT**CCTGAAGCAGCGGGTA	*ppx* (*b2502*) in pET28(a)
**20** LP2-ppx-X1	TTTT**CTCGAG**AGCGGCGATTTCT	
**21** UP-atu0419-E1	GCGC**GAATTC**CATGGCAGTGAGGG	*phoR* (*atu0419*) in pASK-IBA45+
**22** LP-atu0419-H1	GCGC**AAGCTT**TCAAAATCTGACGGTG	
**23** UP-b0400-E1	TTTT**GAATTC**AGTGCTGGAACGGCTG	*phoR* (*eck0394*) in pASK-IBA5
**24** LP-b0400-Sal	AAAA**GTCGAC**ATCGCTGTTTTTGGCAAT	
**25** Eck-SUMO-fw	TTTT**GGTCTC**AAGGTATGGAATTTCACGAAAACAGAGCTAAAGC	*yjiS* (*eck4332*) in pE-SUMO_Amp
**26** Eck-SUMO-rv	TTTT**GGTCTC**ACTAGTCACTCCACATCCTCCCTGCG	
**27** UP-atu1667-B1	TTTT**GGATCC**ATGAACATTGCACGCT	*sdp1* in pKT25/ pKNT25/ pUT18C/ pUT18
**28** LP-atu1667-K1	TTTT**GGTACC**GAAGCCGACGGCGG	
**29** LP-atu1667b-K1	TTTT**GGTACC**TTAGAAGCCGACGGCG	
**30** UP-atu8161-B1	TTTT**GGATCC**ATGAACCCTATCCGCATC	*sdp2* in pKT25/ pKNT25/ pUT18C/ pUT18
**31** LP-atu8161-K1	TTTT**GGTACC**GCGGAACGAACGGGAA	
**32** LP-atu8161b-K1	TTTT**GGTACC**TTAGCGGAACGAACGGGA	
**33** UP-L4-B1	TTTT**GGATCC**ATGAACGTAACACGCAG	*sdp3* in pKT25/ pKNT25/ pUT18C/ pUT18
**34** LP-L4-K1	TT**GGTACC**CTTGCCGACGGCGGCG	
**35** LP-L4b-K1	TTTT**GGTACC**TTACTTGCCGACGGCG	
**36** UP-atu0419-B1	TTTT**GGATCC**ATGGCAGTGAGGGAAGGC	*atu0419* in pKT25/ pKNT25/ pUT18C/ pUT18
**37** LP-atu0419-K1	TTTT**GGTACC**AAATCTGACGGTGAAGTCCG	
**38** LP-atu0419-K2	TTTT**GGTACC**TCAAAATCTGACGGTGAAGTC	
**39** UP-eck0394-B1	TTTT**GGATCC**GTGCTGGAACGGCTGT	*eck0394* in pKT25/ pKNT25/ pUT18C/ pUT18
**40** LP-eck0394-K1	TTTT**GGTACC**ATCGCTGTTTTTGGCAATTA	
**41** LP-eck0394b-K1	TTTT**GGTACC**TTAATCGCTGTTTTTGGCAA	
**42** UP-eck4332-B1	TTTT**GGATCC**ATGGAATTTCACGAAAACAGAG	*eck4332* in pKT25/ pKNT25/ pUT18C/ pUT18
**43** LP-eck4332-K1	TTTT**GGTACC**CTCCACATCCTCCCTGCGTAAC	
**44** LP-eck4332b-K1	TTTT**GGTA**CCTCACTCCACATCCTCCCTGC	

Table S4. Fragments ordered from Twist Bioscience used in this study.

Fragment name	Sequence	Designated use
YjiS_E.coli	TTTTCTCGAGAACCCTCTATAGCCACCAAAAGCTCATAAATTGG<br>TCACAAATTATGCATGAATCACCGCAATCGGGTCAATTTACCGG<br>CTTGCCTTGCGTTTCGTGCATAGGTGACATCTTGCTTTGTCTGT<br>TTTTGTTTCGCTTAAACCGATTATATTCATGGTCATACAGAGATC<br>AGTTCAGCCCCGGACCAAAGCCTCCCAAGACTGGCCGCAGAG<br>ACTGAACAGCACGAAGAGGAAGATGAAAATGGAATTTCACGAA<br>AACAGAGCTAAAGCGCCGTTTATCGGCCTGGTGCAACTCTGGC<br>AGGCGGTGAGGCGTTGGCGGCGGCAAATGCAGACCCGACGC<br>GTGTTACAGCAGATGAGTGATGAGCGGTTGAAGGATATCGGGT<br>TACGCAGGGAGGATGTGGAGGACTACAAAGACCATGACGGTG<br>ATTATAAAGATCATGATATCGACTACAAAGATGACGACGATAAA<br>TGACTCGAGTTTT	*eck4332*^{3xFLAG} from *E. coli* with the upstream region from *sdp1* for pLT4 and pLT5
RSP_6037	TTTTCTCGAGAACCCTCTATAGCCACCAAAAGCTCATAAATTGG<br>TCACAAATTATGCATGAATCACCGCAATCGGGTCAATTTACCGG<br>CTTGCCTTGCGTTTCGTGCATAGGTGACATCTTGCTTTGTCTGT<br>TTTTGTTTCGCTTAAACCGATTATATTCATGGTCATACAGAGATC<br>AGTTCAGCCCCGGACCAAAGCCTCCCAAGACTGGCCGCAGAG<br>ACTGAACAGCACGAAGAGGAAGATGAAAATGGCTTACGCAAAC<br>ACCACCCGTATCGGGCACCACGGTCTGGGAGACCGTGTTTCG<br>GCCCTGGTGGCTTCGGTGAAACTCGCTCTCGCGCAGCGCCGG<br>ATCTACCGGCAGACCGTGCGCGAACTGAACTCGCTGACGACC<br>CGCGAACTGTCCGACCTCGGCATCCACCGCTCGATGATCACC<br>CGTATCGCGATGGAAGCCGCCTACGGTCTCGACTACAAAGAC<br>CATGACGGTGATTATAAAGATCATGATATCGACTACAAAGATGA<br>CGACGATAAATGACTCGAGTTTT	*rsp_RS03480*^{3xFLAG} from *R. sphaerhoides* with the the upstream region from *sdp1* for pLT8
SM2011_RS33625	TTTTCTCGAGAACCCTCTATAGCCACCAAAAGCTCATAAATTGG<br>TCACAAATTATGCATGAATCACCGCAATCGGGTCAATTTACCGG<br>CTTGCCTTGCGTTTCGTGCATAGGTGACATCTTGCTTTGTCTGT<br>TTTTGTTTCGCTTAAACCGATTATATTCATGGTCATACAGAGATC<br>AGTTCAGCCCCGGACCAAAGCCTCCCAAGACTGGCCGCAGAG<br>ACTGAACAGCACGAAGAGGAAGATGAAAATGAACCTCGCACGT<br>TCTTTCAACAACTGGCGCAAGTATCGTCAGACCTGCAACGAAC<br>TCGGCCGCATGAGCGACCGTGAGCTGACCGATCTCGGCATCG<br>GCCGCGCAGACATCCCCTACGTTGCCCGTCAGGCAATCAAGG<br>ACTACAAAGACCATGACGGTGATTATAAAGATCATGATATCGAC<br>TACAAAGATGACGACGATAAATGACTCGAGTTTT	*sm2011_RS33625*^{3xFLAG} from *S. meliloti* with the the upstream region from *sdp1* for pLT14 and pLT15

References

Anschutz P & Deborde J. Spectrophotometric determination of phosphate in matrices from sequential leaching of sediments. *Limnology and Oceanography: Methods* 2016; 14(4), 245–256. doi: 10.1002/lom3.10085

Bachmann BJ. Pedigrees of some mutant strains of *Escherichia coli* K-12. *Bacteriol Rev.* 1972 Dec;36(4):525-57. doi: 10.1128/br.36.4.525-557.1972. PMID: 4568763; PMCID: PMC408331.

Karimova G, Pidoux J, Ullmann A & Ladant D. A bacterial two-hybrid system based on a reconstituted signal transduction pathway. *Proc Natl Acad Sci U S A.* 1998 May 12;95(10):5752-6. doi: 10.1073/pnas.95.10.5752. PMID: 9576956; PMCID: PMC20451.

Karimova G, Ullmann A & Ladant D. Protein-protein interaction between *Bacillus stearothermophilus* tyrosyl-tRNA synthetase subdomains revealed by a bacterial two-hybrid system. *J Mol Microbiol Biotechnol.* 2001 Jan;3(1):73-82. PMID: 11200232.

Schäfer A, Tauch A, Jäger W, Kalinowski J, Thierbach G & Pühler A. Small mobilizable multi-purpose cloning vectors derived from the *Escherichia coli* plasmids pK18 and pK19: selection of defined deletions in the chromosome of *Corynebacterium glutamicum*. *Gene*. 1994 Jul 22;145(1):69-73. doi: 10.1016/0378-1119(94)90324-7. PMID: 8045426.

Studier FW & Moffatt BA. Use of bacteriophage T7 RNA polymerase to direct selective high-level expression of cloned genes. *J Mol Biol.* 1986 May 5;189(1):113-30. doi: 10.1016/0022-2836(86)90385-2. PMID: 3537305.

Torres-Quesada O, Millán V, Nisa-Martínez R, Bardou F, Crespi M, Toro N & Jiménez-Zurdo JI. Independent activity of the homologous small regulatory RNAs AbcR1 and AbcR2 in the legume symbiont *Sinorhizobium meliloti. PLoS One*. 2013 Jul 15;8(7):e68147. doi: 10.1371/journal.pone.0068147. PMID: 23869210; PMCID: PMC3712013.
